# Mutation in Arabidopsis mitochondrial Pentatricopeptide repeat 40 gene affects tolerance to water deficit

**DOI:** 10.1007/s00425-024-04354-w

**Published:** 2024-03-01

**Authors:** Kamal Kant, Gábor Rigó, Dóra Faragó, Dániel Benyó, Roland Tengölics, László Szabados, Laura Zsigmond

**Affiliations:** 1 Institute of Plant Biology, HUN-REN Biological Research Centre, Temesvári Krt. 62, 6726 Szeged, Hungary; 2 Institute of Biochemistry, HUN-REN Biological Research Centre, Temesvári Krt. 62, 6726 Szeged, Hungary

**Keywords:** Arabidopsis, Drought tolerance, Mitochondria, Mutant analysis, Oxidative stress, Pentatricopeptide repeat 40, Water deficit

## Abstract

**Main Conclusion:**

The Arabidopsis Pentatricopeptide repeat 40 (PPR40) insertion mutants have increased tolerance to water deficit compared to wild-type plants. Tolerance is likely the consequence of ABA hypersensitivity of the mutants.

**Abstract:**

Plant growth and development depend on multiple environmental factors whose alterations can disrupt plant homeostasis and trigger complex molecular and physiological responses. Water deficit is one of the factors which can seriously restrict plant growth and viability. Mitochondria play an important role in cellular metabolism, energy production, and redox homeostasis. During drought and salinity stress, mitochondrial dysfunction can lead to ROS overproduction and oxidative stress, affecting plant growth and survival. Alternative oxidases (AOXs) and stabilization of mitochondrial electron transport chain help mitigate ROS damage. The mitochondrial Pentatricopeptide repeat 40 (PPR40) protein was implicated in stress regulation as *ppr40* mutants were found to be hypersensitive to ABA and high salinity during germination. This study investigated the tolerance of the knockout *ppr40-1* and knockdown *ppr40-2* mutants to water deprivation. Our results show that these mutants display an enhanced tolerance to water deficit. The mutants had higher relative water content, reduced level of oxidative damage, and better photosynthetic parameters in water-limited conditions compared to wild-type plants. *ppr40* mutants had considerable differences in metabolic profiles and expression of a number of stress-related genes, suggesting important metabolic reprogramming. Tolerance to water deficit was also manifested in higher survival rates and alleviated growth reduction when watering was suspended. Enhanced sensitivity to ABA and fast stomata closure was suggested to lead to improved capacity for water conservation in such environment. Overall, this study highlights the importance of mitochondrial functions and in particular PPR40 in plant responses to abiotic stress, particularly drought.

**Supplementary Information:**

The online version contains supplementary material available at 10.1007/s00425-024-04354-w.

## Introduction

Plants are in dynamic and complex relationships with their environment, and they rely on essential resources such as light, water, nutrients, and gases, which determine their growth and development. Extreme environmental conditions can limit plant growth by triggering profound physiological and molecular changes. In response to abiotic stresses, plants have developed various mechanisms to cope with the challenges, adapt to the disadvantageous conditions, and survive. Adaptation to changes in environmental conditions requires modulation of metabolic status in order to maintain cellular homeostasis. Such adaptation relies on a complex signaling network which coordinates profound changes in gene expression, and production of protective enzymes and compounds such as antioxidants (Zhu 2016; Takahashi et al. 2018). Among abiotic stresses, drought is widely recognized as one of the most severe environmental constraints influencing the geographic distribution of plants, limiting agricultural productivity and posing a formidable threat to global food security (Hammer et al. 2021). Enhanced osmotic pressure is the primary consequence of water-limited environment. Drought limits water availability which generates cellular dehydration leading to detrimental consequences. Depending on the severity and duration of drought, plant growth is reduced, cell division and expansion is stalled, and photosynthesis is ceased. While plants can recover after moderate, temporal drought, severe water limitation leads to dehydration to a degree that plant viability is compromised (Skirycz and Inzé 2010).

Plants have evolved intricate and diverse mechanisms to combat these challenges. The stress hormone abscisic acid (ABA) plays a crucial role in coordination of molecular and physiological responses, triggering complex signaling network to modulate metabolic processes or gene expression profiles (Finkelstein 2013). Among other responses, ABA induces stomatal closure, which effectively reduces water loss by decreasing transpiration rate in water-limited conditions (Munemasa et al. 2015; Agurla et al. 2018; Muhammad Aslam et al. 2022). While stomatal closure can save water during water deprivation, reduced gas exchange has negative effect on photosynthesis as it reduces CO_2_ availability. In higher plants photosynthesis is primary energy source as it can convert light energy to carbohydrates and other organic compounds. Water deficit and high salinity can disrupt photosynthesis through limited gas exchange, increase photorespiration, lead to decreased CO_2_ assimilation, and enhance generation of reactive oxygen species (ROS). Disturbed photosynthetic electron transport generates ROS which can oxidize membrane lipids, proteins, and nucleic acids leading to oxidative stress (Miller et al. 2010). Therefore, limitation of photosynthesis has severe consequences on plant metabolism and viability.

In the absence of light and in non-photosynthetic tissues mitochondrial respiration provides energy to cellular metabolism. Besides ATP production, mitochondrial respiration can interact with numerous cellular metabolic processes including glycolysis, malate/oxaloacetate cycle, modulate redox and energy pools, and generation and scavenging of ROS (Millar et al. 2011). Mitochondrial metabolism was shown to influence photosynthesis and responses to various biotic and abiotic stresses, and control programmed cell death (PCD) (Møller et al. 2021). During water and salt stress mitochondria are important source of ROS, although the amount they generate is smaller than ROS produced in chloroplasts. Water deficit can impair mitochondrial functions also, leading to over-reduction of mitochondrial electron transport chain (mETC) and enhanced ROS production. Complexes I, II, and III of mETC are principal sources of mitochondrial superoxide which can be converted to hydrogen peroxide by superoxide dismutase (Mn-SOD) (Holley et al. 2011). Mitochondrial H_2_O_2_ can damage cellular structures and is principally responsible for triggering PCD. This response involves the activation of permeability transition pores and subsequent release of cytochrome c. When intracellular ROS surpasses the regulatory threshold, a cascading process is triggered in the mitochondria which undergoes functional impairment and degradation leading to of PCD (Miller et al. 2010; Colombatti et al. 2014; Zhan et al. 2014). ROS accumulation can be reduced by various mechanisms, either by stabilizing metabolisms that generate ROS or by promoting ROS scavenging. ROS generation during stress conditions can be influenced by the mETC stability. In stress conditions alternative oxidases (AOXs) can divert electrons from the cytochrome to non-phosphorylating pathway, preventing over-reduction of the electron transport chain and containing ROS production and damage. AOX can therefore contribute to metabolic and signaling homeostasis and improve tolerance to abiotic stresses (Vanlerberghe 2013).

PPR proteins are characterized by repetitive sequences of approximately 35 amino acids arranged in a tandem array and were identified as principal regulators of RNA editing in mitochondria and chloroplast. PPR genes constitute one of the largest gene families in plants, indicating their functional diversity in controlling organellar metabolism and signaling (Barkan and Small 2014). Other PPR proteins were shown to modulate ABA or ethylene signaling and influence responses to water deprivation or other abiotic stresses (Yuan and Liu 2012; Zhu et al. 2014). The cytosol and nucleus-localized SOAR1 was reported to be implicated in ABA signaling and contribute to the plant's ability to withstand drought, salt, and cold stress (Jiang et al. 2015). GENOMES UNCOUPLED 1 (GUN1) is a chloroplast-localized PPR protein which regulates chloroplast to nucleus retrograde signaling and integrates several developmental and stress-related signals (Koussevitzky et al. 2007). The *gun1* mutant has reduced superoxide dismutase (SOD) and ascorbate peroxidase (APX) activities, enhanced ROS content, and oxidative damage (Fortunato et al. 2022). GUN1 seems to be involved in protection of chloroplasts from oxidative stress. In rice, the mitochondrial PPR protein OsNBL3 is involved in nad5 splicing and is needed for mETC Complex I activity. The nbl3 mutant had elevated alternative respiration; displayed growth retardation, wilting leaves, and premature senescence; and enhanced resistance against several pathogens and tolerance to salt stress (Qiu et al. 2021). OsNBL3-regulated mitochondrial functions are therefore important in stress responses.

The PPR40 protein is associated with Complex III in the mitochondrial electron transport system and was suggested to contribute to sustain mitochondrial electron flow in salt-stressed plants (Zsigmond et al. 2008, 2012). Two insertion mutants of the *PPR40* gene have previously been identified: *ppr40-1* and *ppr40-2* alleles, which were suggested to represent knockout and knockdown mutations, respectively. In germination assays *ppr40-1* and *ppr40-2* displayed stronger and weaker ABA hypersensitivity, respectively, suggesting that the two alleles influence ABA signaling to different degrees. Electron transport of the *ppr40-1* mutant was particularly sensitive to perturbations which resulted in enhanced ROS production, modulating ABA signals and sensitivity to this hormone (Zsigmond et al. 2008). Overexpression of PPR40 improved germination in vitro under salt stress and reduced ABA sensitivity (Zsigmond et al. 2012). While the *ppr40-1* mutant was thoroughly characterized in vitro, phenotype of the *ppr40* mutant alleles and their responses to water deficit in soil-grown plants were not investigated. Here, we show that two mutant alleles of *PPR40* gene *ppr40-1* and *ppr40-2* are more tolerant to gradual water stress, have better capacity to preserve water, reduce oxidative damage, and survive water deprivation.

## Materials and methods

### Plant material and stress treatments

The T-DNA insertion mutants *ppr40-1* and *ppr40-2* of *Arabidopsis thaliana* with Col-0 background have previously been characterized (Zsigmond et al. 2008). The plants were grown on Plantobalt Substrate 1 sieved soil (https://www.plantaflor.de/en/products/propagation/details/plantobalt-substrate-1-fine-80-20-clay). Plants were grown in growth chambers (FitoClima, Aralab, Portugal), under controlled culture conditions at 22 °C, 8/16 h light/dark cycle, ~ 150 µmol m^−2^ s^−1^ photon flux density provided by fluorescent tube illumination. Plants were grown for 4 weeks, and then water stress was applied by withholding the water for 10 days. For recovery, plants were re-watered and cultured for additional 10 days in standard growth conditions. To determine the recovery rate, the number of healthy, green plants was counted and the percentage of surviving plants was calculated.

### Chlorophyll fluorescence measurement

Photosynthetic capacity was evaluated in plants cultured in growth chambers (see above) subjected to water stress for 10 days. Electron transport rate (ETR) was measured using Imaging-PAM (M-Series, Maxi version; Heinz Walz GmbH) as described (Baker 2008). Prior to imaging, plants were adapted to darkness for 15 min. ETR was determined using a rapid light curve, which involved measuring fluorescence parameters at different photosynthetic photon flux densities of 0, 55, 110, 185, 280, 335, and 395 µmol m^−2^ s^−1^. Two leaf areas were selected on each plant, and a total of 15 plants were measured for each genotype. The experiment was repeated three times.

### Measurement of stomata conductance

Stomata conductance of Col-0 wild-type and *ppr40-1* mutant plants was measured with LI-6400 portable photosynthesis system (LiCor, Lincoln, NE, USA) as described (Hepworth et al. 2015). Relative humidity of the measuring chamber was kept at 65%, the flow rate was 300 µmol s^−1^, and the block temperature was 23 °C. The reference CO_2_ was maintained at 400 ppm, and light intensity at 150 µmol m^−2^ s^−1^. Measurements were made at daily intervals after irrigation was stopped. 15 plants per genotype were analyzed.

### Relative water content

Relative water content (RWC) was measured in plants cultured in growth chamber (see above) after 10 days of water withdrawal. Fresh leaves were collected from approximately 30 individual plants from each plant line. RWC was measured as described (Barrs and Weatherley 1962). The experiment was repeated 3 times.

### Proline estimation

Proline accumulation was tested in plants cultured in growth chambers (see above) and exposed to water stress for 7 to 10 days. Free proline content was determined using a ninhydrin-based colorimetric assay (Kovács et al. 2019). The absorbance of the reaction product was determined spectrophotometrically at 520 nm using Thermo Scientific, Multiscan Go Microplate Spectrophotometer. Proline concentration was calculated with a standard curve. Four replicates were measured for each genotype and treatment. Experiments were repeated three times.

### LC/MS analysis

**Fig. 1:**
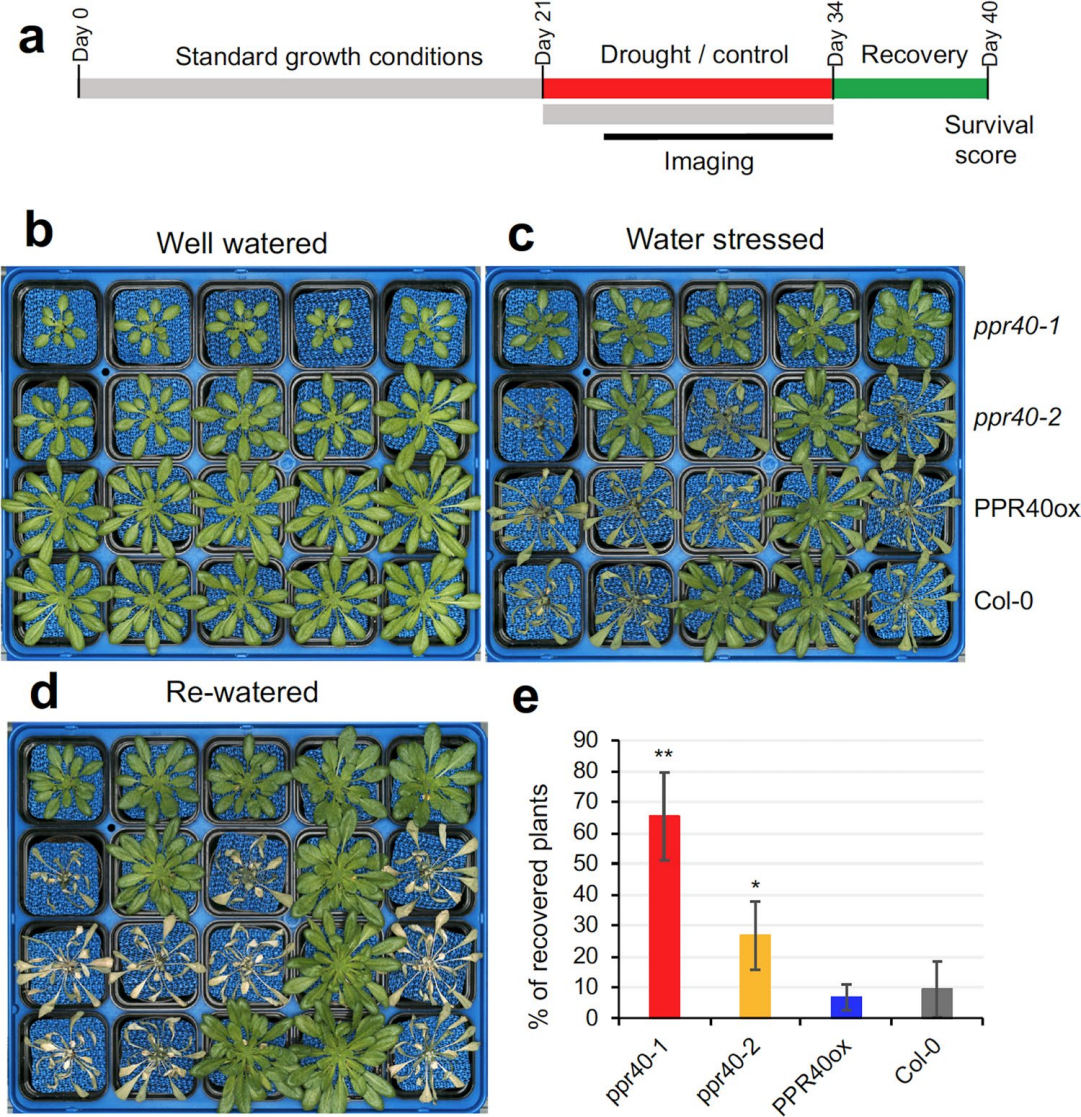
The *ppr40-1* and *ppr40-2* mutants survive water limitation at higher rates than Arabidopsis Col-0 wild-type plants. Plants were cultured in plant phenotyping system. 3-week-old plants were deprived of water for 13 days and subsequently re-watered. **a** Experimental design. **b**-**d** Representative images of each genotype grown in a phenotyping tray. **b** Plants before watering was withdrawn (day 0). **c** Plants after 12 days of water stress. **d** Plants 6 days after re-watering. **e** Survival rates in a typical experiment. 80 plants were monitored in this experiment, scored in groups of 10. Percentage (%) of recovered plants is shown. Error bars indicate standard deviation (*n* = 8). Statistical significance was determined with t-test, * and ** indicate significant differences compared to Col-0 at *P* < 0.05 and *P* < 0.01 levels, respectively

For LC/MS analysis, leaf material was harvested from 4-week-old Col-0 and *ppr40-1* mutant plants, stressed by water deprivation (see Fig. 1a). 50 mg leaf material was collected, weighed, and frozen in liquid nitrogen before grinding with a MM400 laboratory mill (Retsch, Haan, Germany) at 30 Hz for 1 min. After homogenization, the samples were resuspended in extraction buffer (40%, v/v) methanol, 20% (v/v) water, 40% (v/v) acetonitrile. After adding the extraction buffer (1 ml for 30 mg leaves), the samples were vortexed vigorously for 10 min. This was followed by centrifugation at 20.000 g, at 4 ºC, for 10 min and the supernatant was collected.

For metabolome analysis, an ultrahigh-performance liquid chromatography-tandem mass spectroscopy (UPLC–MS/MS) system, namely, a Thermo Q Exactive Focus instrument (Thermo Fisher Scientific, Waltham, MA, USA) equipped with a Dionex Ulimate 3000 UHPLC system was used. For chromatographical separation an XBridge Premier BEH Amide column (2.1 mm × 150 mm, 2.5 μm; Waters Corporation) was used. From each sample 5 μl was injected into the system. The mobile phase composition was gradually changed from 98% solvent A (97.5/2.5 acetonitrile/water to 80% solvent B (20/80 acetonitrile / 20 mM ammonium acetate + 20 mM ammonia in water) in 9 min using a flow of 400 μl/min with gradient profile 6. After 1 min of equilibration the eluent composition returned to the initial conditions in 0.5 min and the system was equilibrated for 4 min. For acquiring semi-quantitative metabolome data, system was operated in MS1 scan mode using negative ionization. Data were acquired using the following settings: sheath gas: 55 l/min, aux gas: 14 l/min, sweep gas: 4 l/min, probe heater 440 °C, and capillary temperature 280 °C with 2500 V capillary voltage in negative ionization mode. Scanning mass range was 80–800 m/z with an AGC target 3*10^6^ and maximum IT time 250 ms. Pooled extract samples as QCs were injected multiple times across the batch following the guidelines of Broadhurst et al. (2018).

For metabolite identification and verification of the six preselected compounds pooled extract samples were injected multiple times at the end of the sequence. For acquiring MS2 data NCE 10, 30, and 50 were used for DDMS confirmation mode.

To obtain peak intensity values, the recorded data were processed using Tracefinder. 37 putatively identified metabolite signal intensities (peak area) were extracted based on their retention time (± 0.25 min) and exact mass data (± 5 ppm). PQN normalization was applied to metabolite data, and 6 metabolites were selected for MS2 level confirmation. Compound Discoverer 2.1 was used for MS2 level putative metabolite identification searching against the endogenous metabolites compound class of mzCloud database. The presented metabolites were identified at the highest available level A (Alseekh et al. 2021). ANOVA analysis was performed with using the aov function of stats package. Statistical analysis and plotting were performed with using tidyverse and ggplot (Wickham et al. 2019). All statistical and normalization procedures were performed using the R statistical language version 4.0. All data will become available in the MetaboLights repository. Analysis was made in 6 repetitions.

### Lipid peroxidation assay

Lipid peroxidation was measured in plants cultured in growth chambers (see above) and exposed to water stress for 7–9 days. Lipid peroxidation was assessed using the thiobarbituric acid-reactive substances (TBARS) assay as described (Heath and Packer 1968). Leaf tissues (100 mg) from three biological replicates of all genotypes and treatments were homogenized in 1 ml of 0.1% trichloroacetic acid (TCA) containing 0.4% butylhydroxytoluene. The homogenates were centrifuged at 15,800 g for 20 min, and 250 μl of the supernatant was mixed with 1 ml of 20% TCA containing 0.5% thiobarbituric acid (TBA). The mixture was then incubated at 96 °C for 30 min. After incubation, the absorbance was measured at 532 nm using a Multiskan GO microplate reader (Thermo Fisher Scientific) and adjusted by subtracting the non-specific absorbance measured at 600 nm. The concentration of malondialdehyde (MDA), a marker of lipid peroxidation, was calculated using the extinction coefficient ε532 = 155 mM^−1^ cm^−1^. The experiment was performed in three biological replicates.

### Gene expression study

Expression of stress-induced marker genes was determined in plants cultured in growth chambers (see above) and exposed to water stress for 8 days. Total RNA was isolated from 100 mg Arabidopsis leaves as described (Yaffe et al. 2012). RNA was treated with TURBO DNA-free™ Kit (Thermo Fisher Scientific) and then 1 µg RNA was used for cDNA Synthesis using the High Capacity cDNA Reverse Transcription Kit (Applied Biosystems). Real-time PCR was carried out with the ABI 7900 Fast Real-Time System (Applied Biosystems) using SYBR Green qPCR Master Mixes (Thermo Scientific) following the manufacturer's instructions. Relative transcript levels were standardized to *UBC18* (*AT5G42990*) and *ACT2* (*AT3G18780*) and calculated by the 2^−∆Ct^ or 2^−ΔΔCt^ method (Livak and Schmittgen 2001). Three replicates were made. Oligonucleotides used in this study are listed in Supplementary Table S1.

### Plant phenotyping

Phenotypic responses of wild-type Col-0 and *ppr40-1* and *ppr40-2* mutants were studied with the PlantScreen™ Compact System (Photon System Instrument [PSI) phenotyping system) following a previously established protocol (Faragó et al. 2022). The plants were grown in individual pots filled with Plantobalt Substrate 1 soil, in controlled environmental conditions (Walk-in FytoScope growth chamber, PSI, Drasov, Czech Republic) under 8 h/16 h light/dark cycle, 22 °C/20 °C temperature, and 65% relative humidity. The illumination was provided by light-emitting diodes (LEDs) with cool white (5700 K), deep red (660 nm, 30%), blue (470 nm, 20%), far red (740 nm, 30%), and ultraviolet (UV) (405 nm, 20%) with a photon irradiance of 130 µmol m^−2^ s^−1^. The plants were grown for 21 days after sowing and then subjected to water stress by suspending watering for 13 days, followed by re-watering for recovery. RGB imaging was made at daily intervals during the dehydration period using a top view GigE PSI RGB camera with 12.36 MP resolution. Chlorophyll fluorescence (ChlF) images were obtained with FluorCam FC-800MF Pulse Amplitude-modulated (PAM) system with a pixel resolution of 1360 × 1024, frame rate of 20 fps, and 16-bit depth. ChlF imaging was performed after 15 min of dark adaptation, following a protocol that included pulse-modulated short duration measuring flashes with red–orange 620 nm light at 33 µs for Fo, saturation pulse with cool-white light at 1200 µmol m^−2^ s^−1^ irradiance in the dark-adapted state for Fm, and 180 s intervals of cool-white actinic light at 130 µmol m^−2^ s^−1^. 40 plants were analyzed for each genotype and treatment. The PSI PlantScreen™ Data Analyzer software was used for image processing and retrieval of raw data, which were further processed using RStudio software. The dynamic responses among genotypes were characterized, and outliers were identified and removed from the curated dataset using a statistical approach of analysis of variance (ANOVA) (Kruskal–Wallis) with pairwise Wilcoxon's test/Mann–Whitney's test of significance with *P* < 0.05, as described previously (Julkowska et al. 2019).

### Software tools

All the data analyses were implemented using software Rstudio (RStudio Team (2020). RStudio: Integrated Development for R. RStudio, PBC, Boston, MA, USA; URL http://www.rstudio.com/) and Microsoft Excel (2016). Statistical analyses (One-way and Two-way ANOVA, followed by Tukey or Fisher’s LSD post hoc test) were performed using the OriginPro 2018 software version 9.5 (OriginLab Corporation, Northampton, MA, USA). The differences between means were determined by Duncan's multiple range test and labeled in diagram with different letters.

## Results

### The *ppr40* mutants display tolerance to water deficiency

PPR40 was previously shown to control mitochondrial electron transport and be implicated in stress responses. In germination assays *ppr40-*1 knockout and the *ppr40-2* knockdown mutants displayed strong and intermediate ABA hypersensitivity, respectively. Enhanced ABA sensitivity resulted in faster stomata closure and reduced evaporation of the *ppr40-1* mutant (Zsigmond et al. 2008). In order to evaluate the consequences of altered ABA responses in water-limited conditions, survival of the two *ppr40* mutants was compared to Col-0 wild-type plants after water stress. Plants were grown in controlled conditions using the plant phenotyping platform. Three-week-old plants were deprived of water for 13 days and re-watered to allow recovery (Fig. 1a). Soil water content dropped after watering was suspended which reduced growth and viability (Figs. 1b–c, S1a). After re-watering, rosettes of surviving plants became green, resumed turgor, and started growing, while dead plants became chlorotic (Fig. 1d). When survival rates were compared, *ppr40-1* mutant plants recovered with significantly higher frequency than wild-type plants. Survival rates of the *ppr40-2* mutant were intermediate, while transgenic plants which overexpressed the PPR40 recovered similarly to wild-type plants (Fig. 1e). Differences in plant survival among the tested genotypes were similar in several independent experiments.

**Fig. 2:**
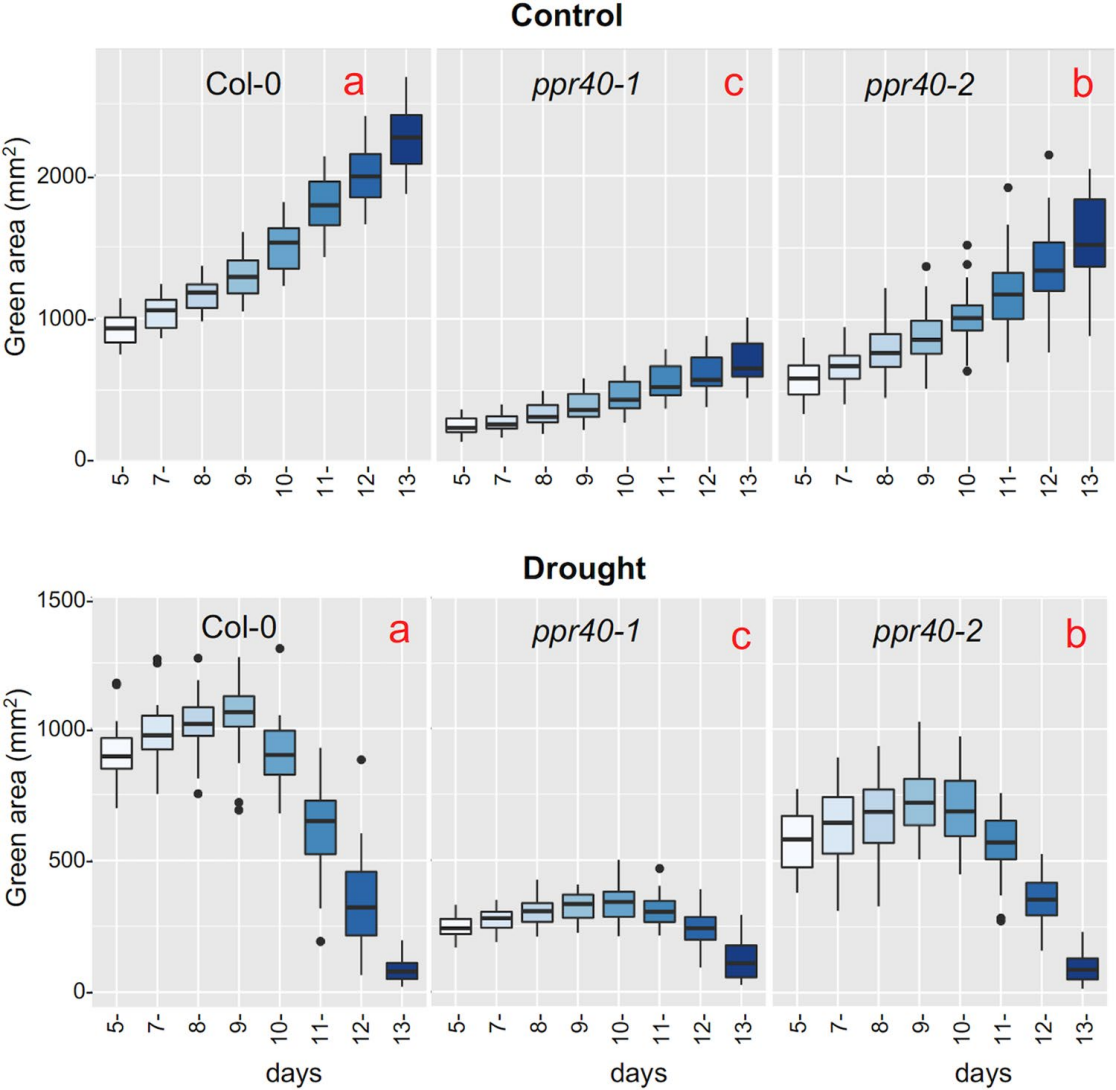
Growth of Arabidopsis plants in standard and water-deprived conditions, as revealed by phenotypic analysis. Plant growth was monitored by analysis of RGB images, obtained between 5 and 13 days after watering was suspended. Change of rosette size was determined by measuring color-segmented green area of 40 plants of each genotype. Control: uninterrupted watering; drought: water withdrawal. Statistical analysis used Kruskal–Wallis test, to compare the treatments and genotypes. Error bars represent the standard deviation of means green area of 40 plants from each genotype. Different letters (in red) indicate significant differences at *P* < 0.05.

Growth and morphology of well-watered and water-stressed plants were compared by periodical imaging in an automatic plant phenotyping platform. RGB images were processed to estimate rosette size as green pixel area or morphological parameters such as slenderness of leaves (SOL). Change in rosette sizes of well-watered plants resembled logarithmic growth, with considerable differences in growth rates. In control conditions *ppr40-1* and *ppr40-2* plants were 70% and 30% smaller than Col-0, respectively (Fig. 1, 2, Fig. S1b). Automatic phenotyping confirmed the semidwarf growth habit reported for these mutants (Zsigmond et al. 2008). Growth of rosette sizes was seriously hampered by water stress. Green area of Col-0 plants grew until day 9 of water withdrawal, then dropped fast, indicating loss of chlorophyll content. Decline in green area of *ppr40-1* mutant started on day 11 after watering was stopped, while it was intermediate in the *ppr40-2* mutant (Fig. 2). Calculation of normalized values for green pixel areas revealed that in well-watered condition growth of relative rosette sizes was similar, while water-deprived Col-0 plants collapsed earlier and faster than the ppr40 mutants. Decline in relative green area of *ppr40-1* was most delayed, while *ppr40-2* was intermediate when compared to Col-0 (Fig. S2). Such normalized parameters could clearly indicate the sustained viability of *ppr40* mutants when compared to wild-type ones in water-limited conditions.

**Fig. 3:**
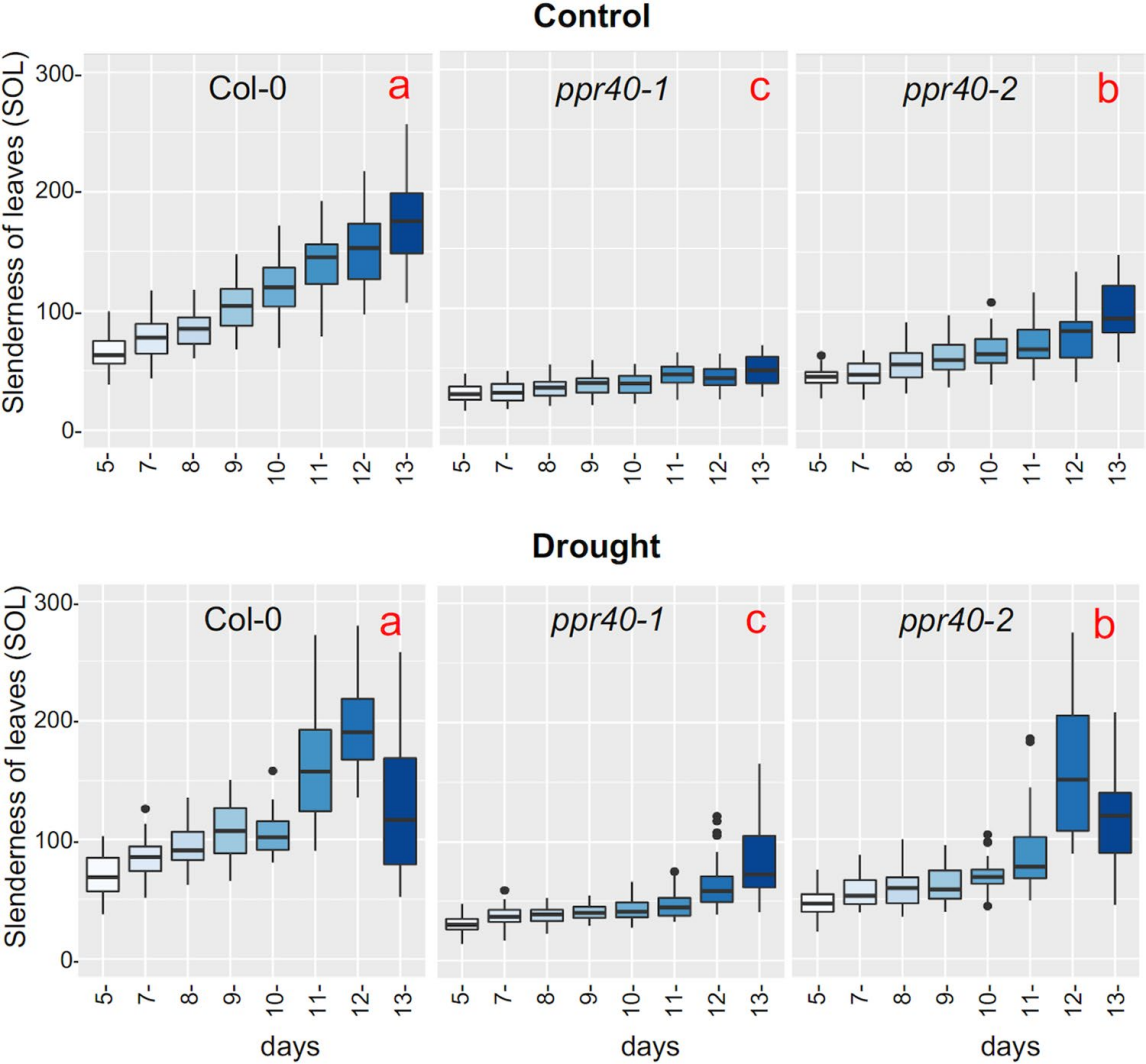
Change of plant morphology as displayed by slenderness of leaves (SOL) parameter. Plant growth was monitored by a phenotyping platform as described in Fig. 2. SOL was calculated from RGB images. Control: uninterrupted watering; drought: water withdrawal. Statistical analysis used Kruskal–Wallis test, to compare the treatments and genotypes. Error bars represent the standard deviation of means green area of 40 plants from each genotype. Different letters (in red) indicate significant differences at *P* < 0.05

Color-segmented RGB images were used to determine hue ratios in well-watered and water-stressed plants (Awlia et al. 2016). Greenness hue indexes were similar in well-watered plants, while genotype-dependent shifts in hue abundances were observed in plants exposed to 12 days of water deprivation (Fig. S3). The parameter slenderness of leaves (SOL) displays the ratio of length and width of leaves and indicates morphological changes during plant development (Pavicic et al. 2017). SOL values gradually increased with time in well-watered plants. In water-stressed Col-0 plants SOL values sharply increased on day 11 and subsequently dropped on day 13, indicating a rapid decline in rosette morphology. Change in SOL values of water-stressed *ppr40-1* mutant was delayed and did not drop in the observation period. Change of SOL in *ppr40-2* mutant was intermediate between Col-0 and *ppr40-1* (Fig. 3). These data suggest that plant morphology is better preserved in the *ppr40-1* mutant in water-limited conditions.

### Physiological parameters confirm tolerance of ppr40 mutants to water deficiency

**Fig. 4:**
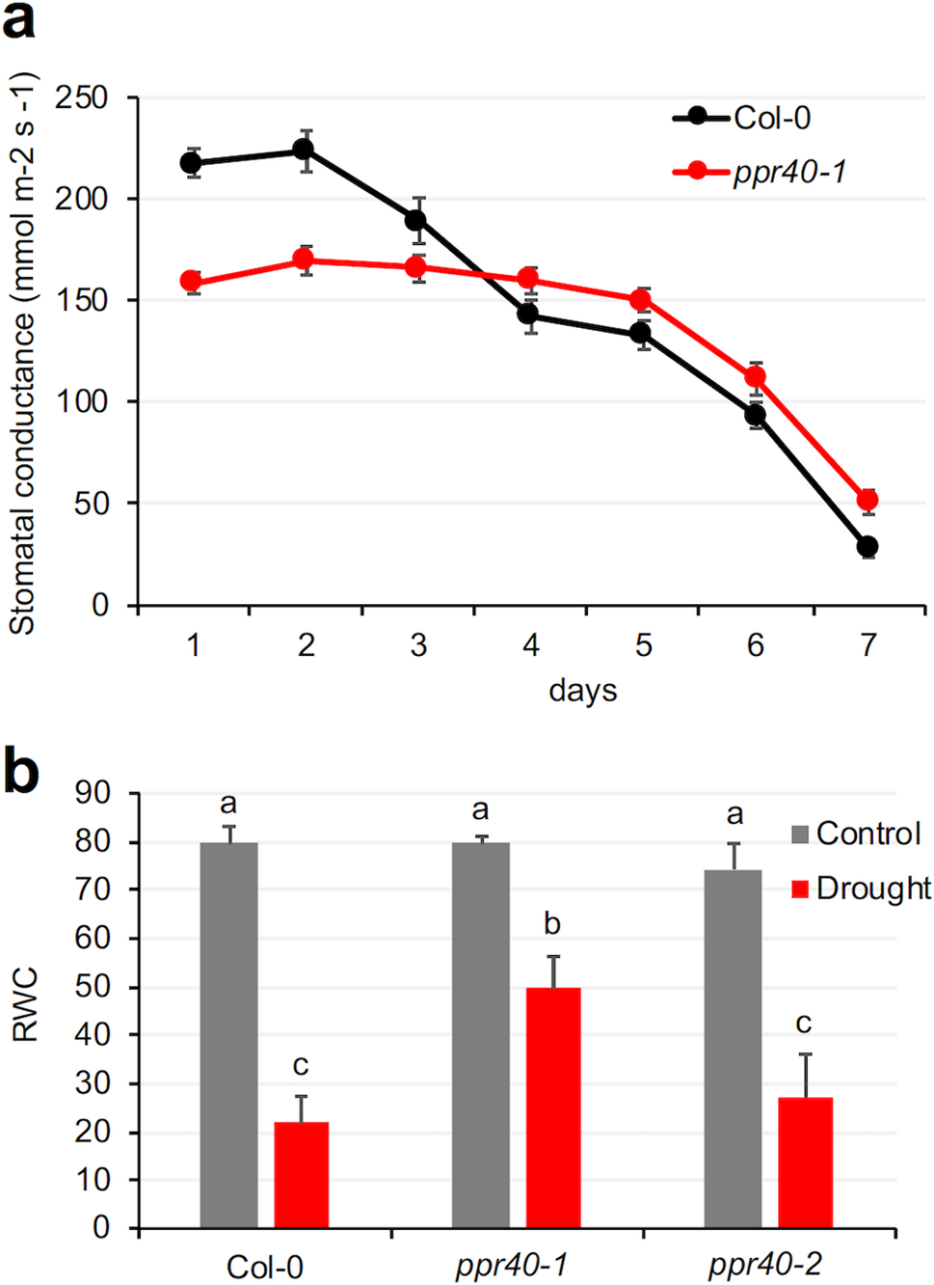
Effect of water deprivation on stomatal conductance and Relative Water Content (RWC). **a** Change of stomatal conductance of Arabidopsis Col-0 wild-type and *ppr40-1* mutant plants. Four-week-old plants were deprived of water for 7 days, and stomatal conductance was measured at daily intervals. **b** RWC in Arabidopsis *ppr40-1* and *ppr40-2* mutants and Col-0 wild-type plants, subjected to water deprivation for 10 days. Error bars indicate standard deviation. Experiments were performed in a growth chamber and repeated three times. Statistical analysis used Two-way ANOVA, Tukey test. Different letters indicate significant differences at *P* < 0.05

Enhanced ABA sensitivity can lead to faster stomata closure, which helps plants preserve water during water shortages. Stomatal conductance indicates gas exchange and transpiration, which is determined by stomata aperture. ABA hypersensitivity of the *ppr40-1* mutant correlated with faster stomata closure; therefore, stomata conductance was compared in water-stressed wild-type and mutant plants. Stomatal conductance of the *ppr40-1* mutant was lower than Col-0, in well-watered conditions and up to three days after watering was suspended. While stomatal conductance was initially higher in wild-type plants than in *ppr40-1* mutant, it dropped faster in water-restricted conditions. Under severe water deprivation stomata conductance of both genotypes became considerably reduced (Fig. 4a).

Relative water content (RWC) is one of the key physiological features that is used to describe cellular hydration and consequences of water deficit (Barr and Weatherley 1962). In order to get information about water status of stressed plants, RWC of wild-type plants and *ppr40* mutants was determined in fully hydrated and dehydrated plants. RWC in well-watered plants was similar among the tested genotypes. Water stress led to 75% reduction of RWC in Col-0 plants, which was reduced by 40% and 63% in *ppr40-1* and *ppr40-2* mutants, respectively (Fig. 4b). These data indicate that more water was retained in the water-stressed *ppr40-1* mutant than in Col-0 (*ppr40-2* was intermediate), which correlated with slower wilting and higher viability of these plants (Figs. 1–3).

**Fig. 5:**
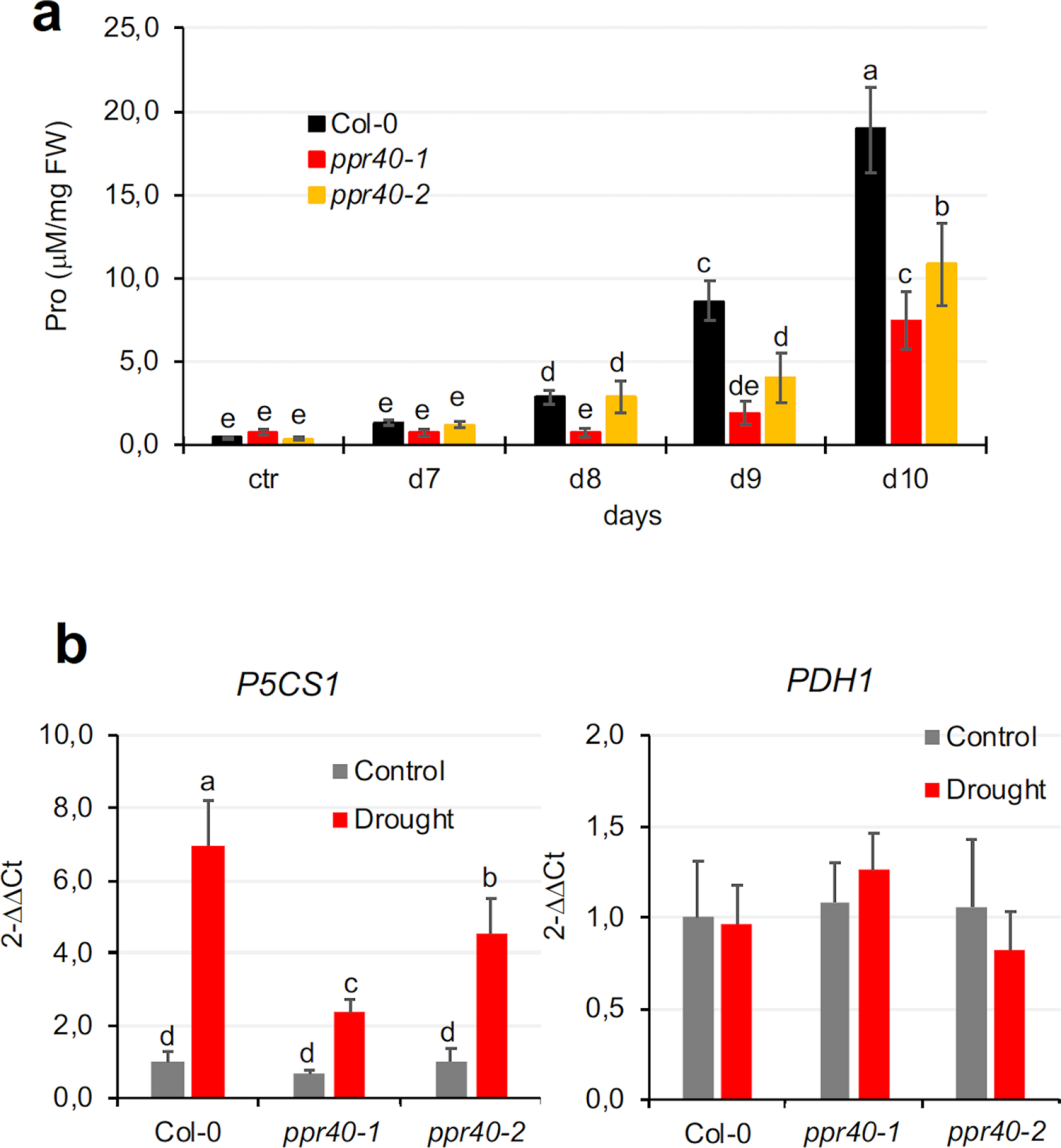
Proline accumulation and expression of *P5CS1* and *PDH1* genes in *ppr40-1, ppr40-2* mutants, and wild-type (Arabidopsis Col-0) plants in response to water deprivation. Experiments were performed in a growth chamber. **a** Change in proline content of wild-type and mutant plants which were stressed by water withdrawal for up to 10 days. **b** Expression of *P5CS1* and *PDH1* genes in water-stressed and well-watered plants after 8 days of treatment. Relative expression is shown where 1 corresponds to transcript levels of non-treated Col-0 plants. Statistical analysis used Two-way ANOVA, Fisher’s LSD, and Tukey test. The error bars indicate the standard deviation of means of three biological replicates. Different letters indicate significant differences at *P* < 0.05

Proline accumulation is a well-known physiological response to water deprivation in higher plants and is related to defenses during osmotic and oxidative stresses (Alvarez et al. 2022). In standard growth conditions, proline content of *ppr40-1* was slightly higher, while *ppr40-2* was similar to Col-0 wild-type plants (Fig. 5a). Proline levels of Col-0 and *ppr40-2* plants started to increase after 7 days of water deprivation. In contrast, there was little change in proline content of the *ppr40-1* mutant until 8 days of water stress, which increased at limited rate afterward. On 10th day of water withdrawal, proline content of *ppr40-1* and *ppr40-2* mutants was 60% and 40% lower than in wild-type plants, respectively. Kinetics of proline accumulation in *ppr40-2* was slower than that in Col-0 but faster than that in *ppr40-1*, correlating with the intermediate phenotype of this mutant (Fig. 5a).

Proline metabolism is regulated by two key enzymes, the ∆-pyrroline carboxylate synthase (P5CS) and the proline dehydrogenase (PDH), controlling the first steps of biosynthesis and catabolism, respectively (Szabados and Savouré 2010). In Arabidopsis, both enzymes are encoded by two genes with differences in their transcriptional regulation. *P5CS1* is an ABA-regulated gene which is essential for stress-dependent proline accumulation (Székely et al. 2008). *P5CS1* expression was enhanced by 7 times in water-stressed wild-type plants, while three to five times enhancements were detected in *ppr40-1* and *ppr40-2* mutants, respectively (Fig. 5b). Expression of the *PDH1* gene did not change in these conditions and was similar in the wild-type and mutant plants (Fig. 5b). These results indicate that diminished *P5CS1* gene activation and inferior proline biosynthesis are responsible for reduced proline accumulation in the *ppr40* mutants in water-limited conditions.

### Metabolomics

**Fig. 6:**
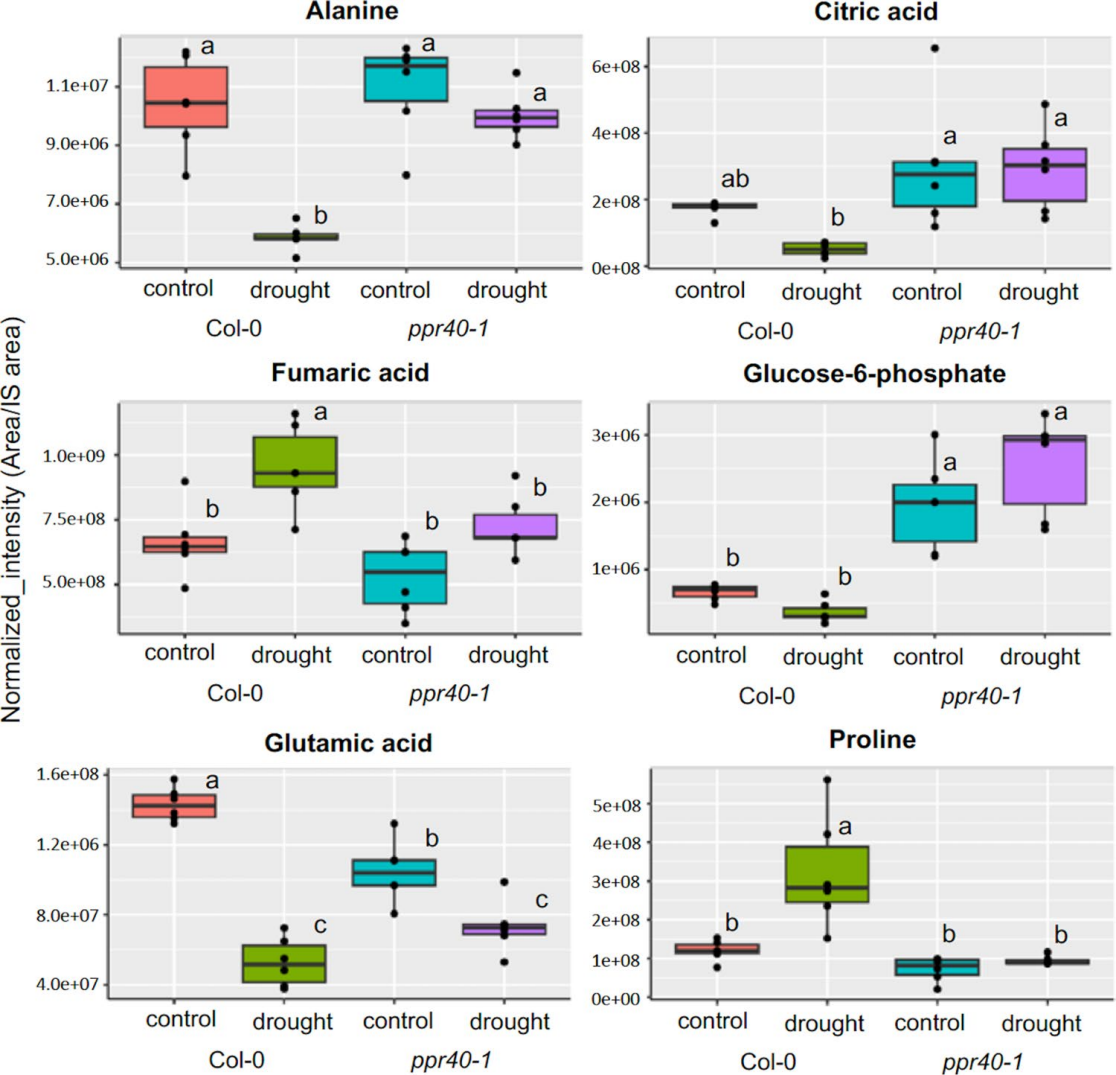
Metabolic profiles of six selected metabolites in Col-0 wild-type and *ppr40-1* mutant plants. Metabolite profiles were determined by LC/MS analysis in leaves of four-week-old plants, cultured in well-watered (control) and water-restricted (drought) conditions. Statistical analysis used Two-way ANOVA and error bars represent the standard deviation of means of 6 samples of each genotype

Besides having an essential role in energy supply and redox control, plant mitochondria are important metabolic powerhouses which influence several important metabolic pathways through respiration (Millar et al. 2011). In order to decipher the consequences of the *ppr40-1* mutation in mitochondrial metabolism a metabolomic survey was performed, targeting mainly TCA cycle, sugar, and amino acid metabolic pathways. Water deprivation led to significant alterations in concentrations of various organic acids of the TCA cycle and amino acids which were less affected in the *ppr40-1* mutant (Fig. 6). Alanine and glutamate contents were considerably reduced by water deprivation in Col-0 plants and were only moderately abated in *ppr40-1*. Differences in proline accumulation could be observed in the metabolomic survey also: when compared to wild-type plants, proline accumulation was diminished in water-stressed *ppr40-1* mutant. In wild-type plants concentrations of citric acid and glucose-6-phosphate were reduced by water stress, but were elevated in *ppr40-1* with or without water deprivation. Fumaric acid content was lower in *ppr40-1* than in wild-type plants and was only moderately enhanced by drought in this mutant. These results demonstrate that various metabolic pathways were altered *ppr40-1* mutant, including the mitochondrial TCA cycle.

### Photosynthesis is more stable in *ppr40* mutants during water deficiency

**Fig. 7:**
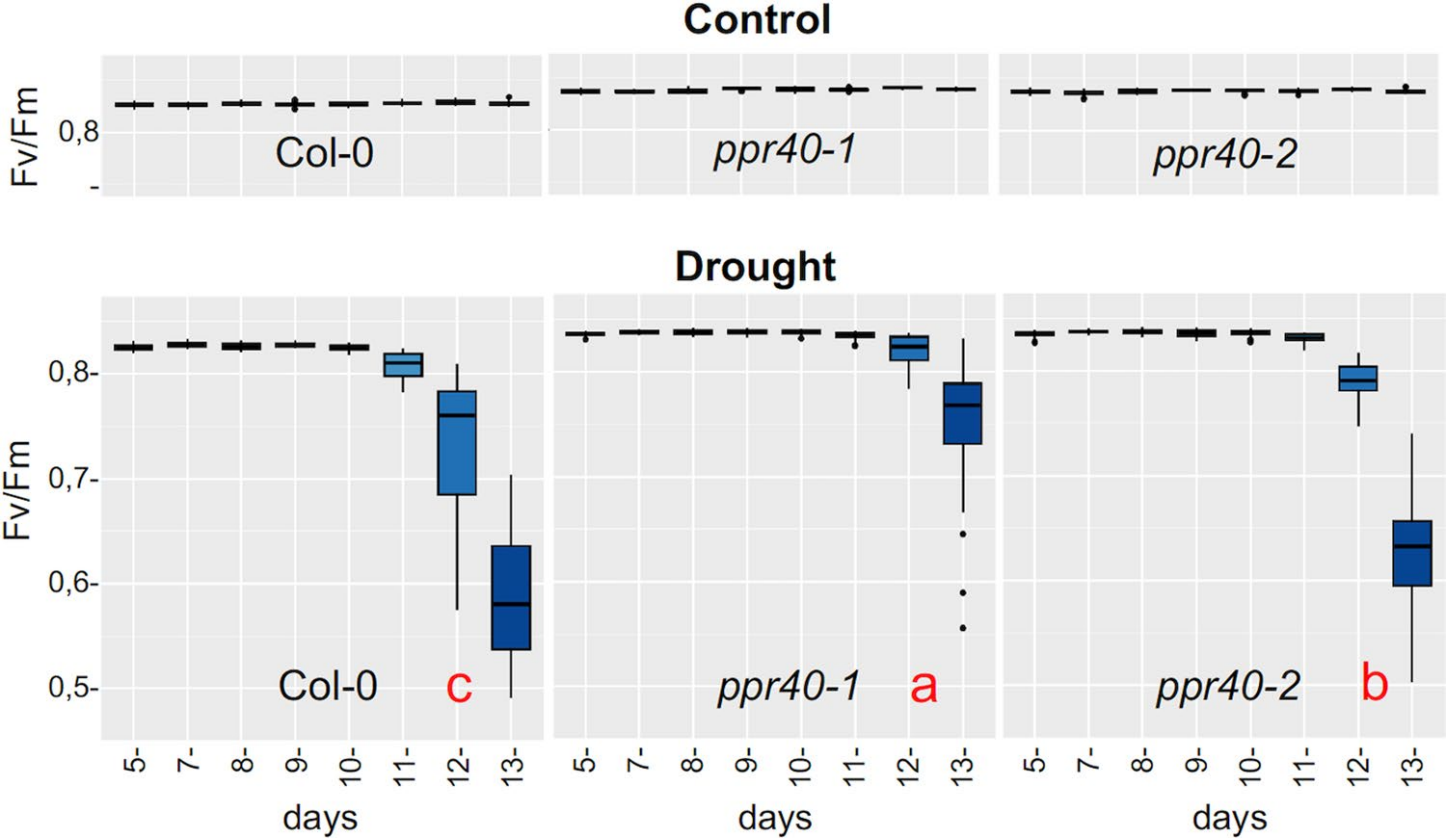
The impact of water deprivation on the photosynthetic parameters of Arabidopsis Col-0 wild-type and *ppr40* mutant plants. Chlorophyll fluorescence (ChlF) imaging was used to monitor changes of photosynthetic parameters of water-stressed and well-watered plants using the automatic plant phenotyping platform. Experimental design is shown in Fig. 1a. Change of the maximum quantum yield of photosystem II (Fv/Fm) is shown (averages of 40 plants). Control: uninterrupted watering; drought: water withdrawal. Statistical analysis used Kruskal–Wallis test, to compare the treatments and genotypes. Error bars represent the standard deviation of means Fv/Fm of 40 plants from each genotype. Different letters (in red) indicate significant differences at *P* < 0.05

Photosynthesis is a drought-sensitive physiological parameter in plants. To characterize the changes in photosynthesis in water-stressed mutants, chlorophyll fluorescence was monitored by phenotyping image analysis in plants cultured in standard or water-deprived conditions. The maximum quantum yield of PSII photochemistry (expressed as Fv/Fm) was similar in wild-type and mutant plants during standard growth conditions, showing values of 0.83–0.84 during the observation period. When watering was suspended, Fv/Fm values were similar to control in Col-0 plants until 10th day of water deprivation, then sharply dropped afterward reaching an average 0.57 on day 13. Fv/Fm in the *ppr40-1* mutant was more stable in such conditions and started declining on 12th day after watering was stopped. Average Fv/Fm value of *ppr40-1* was still around 0.77 on day 13. Change in Fv/Fm of *ppr40-2* mutant was intermediate, it dropped faster than in *ppr40-1*, but decreased later than in Col-0, as it started to decline on day 12 (Fig. 7). Image-based analysis of chlorophyll fluorescence in time could reveal significant differences between the wild-type and *ppr40* mutant plants in maximum quantum yield of PSII, which is one of the most commonly used photosynthetic parameters in plant stress biology (Fig. S4). Differences in kinetics of Fv/Fm values indicated that photosynthetic electron transport remained functional for more extended periods in the water-stressed *ppr40* mutants than in wild-type plants.

**Fig. 8:**
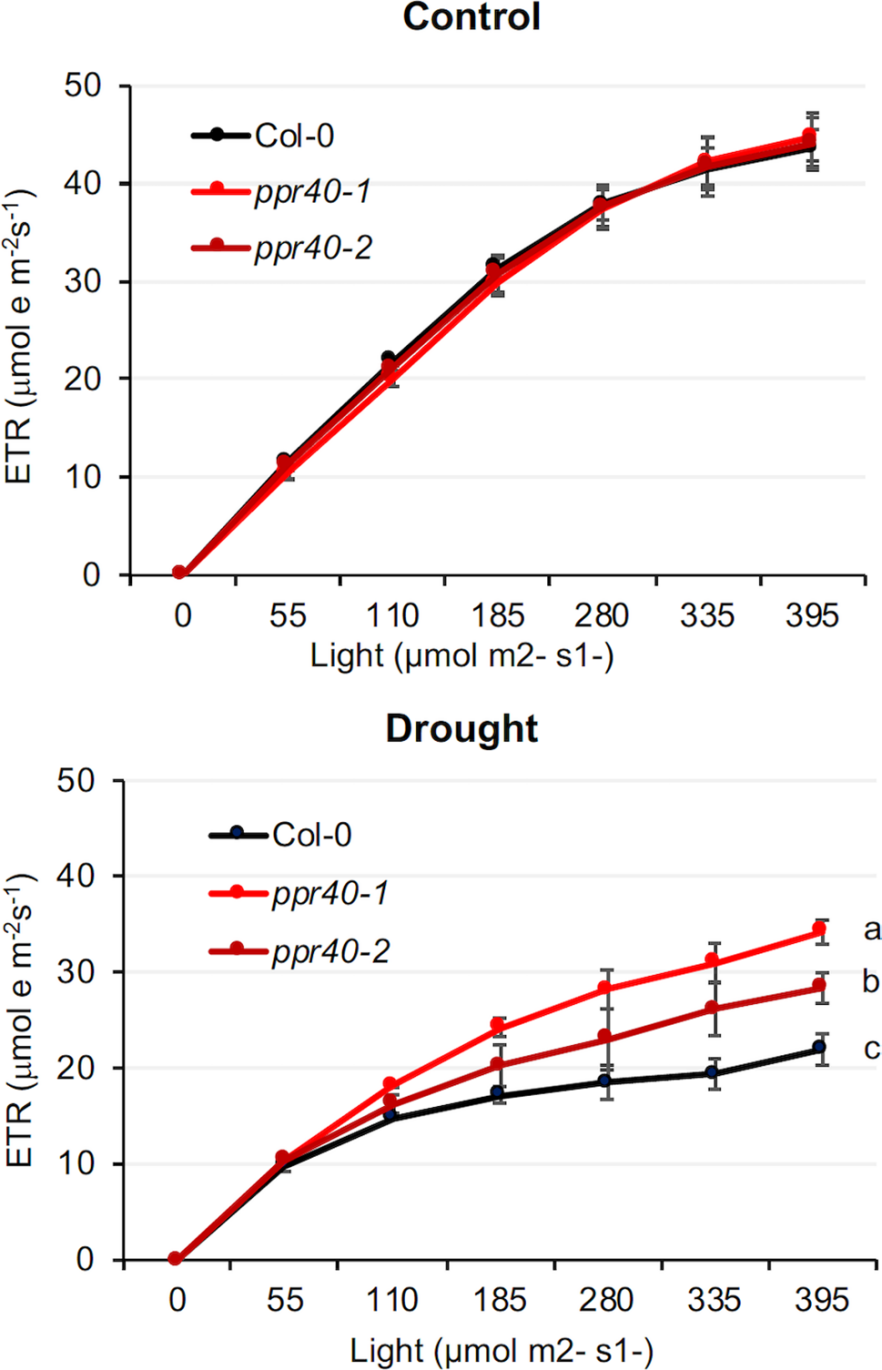
Quantitative analysis of electron transport rates (ETR(II) in *ppr40-1* and *ppr40-2* mutants, and in wild-type (Arabidopsis Col-0) plants. ETR(II) was measured in dark-adapted conditions using a pulse amplitude-modulated fluorometer (Maxi-PAM). ETR values were obtained from well-watered (control) and water-stressed plants deprived of water for 10 days (drought). Error bars indicate standard deviation (*n* = 3). Experiments were repeated three times. Statistical analysis used Two-way ANOVA, Tukey test. Different letters indicate significant differences at *P* < 0.05

Differences in maximum quantum yield of PSII prompted us to study the photosynthetic electron transport in the mutants in more detail. Electron transport rates (ETR) were analyzed in separate experiments using plants cultured in growth chambers under well-watered and water-deprived conditions. ETR(II) values were similar in wild-type and *ppr40* mutants when plants were cultured in standard conditions. In these conditions, 10 days of water deprivation reduced ETR(II) by 50 to 60% in Col-0 wild-type plants. Such reduction in ETR(II) was, however, significantly alleviated in the *ppr40-1* mutant, which had 35% higher ETR(II) than the water-stressed Col-0 plants. ETR(II) values in *ppr40-2* were intermediate between Col-0 and *ppr40-1* in water-stressed conditions (Fig. 8). Our results indicate that photosynthesis is less affected by water stress in *ppr40* mutants than in wild-type Arabidopsis plants, and the difference between the knockout and knockdown mutants correlates with phenotypic changes and other physiological data.

### Oxidative damage is alleviated by ppr40 mutations

**Fig. 9:**
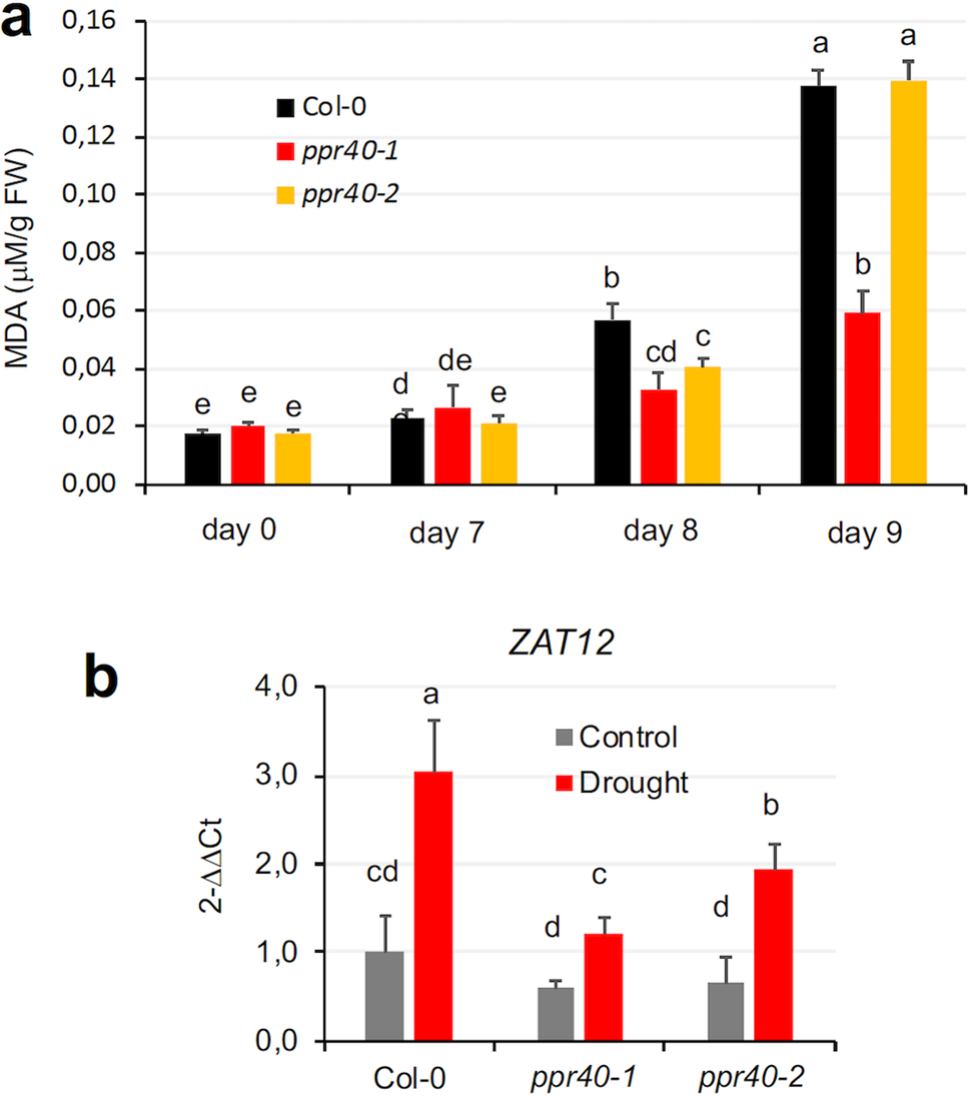
Oxidative damage in water-deprived plants. **a** Lipid peroxidation rates of Arabidopsis Col-0 wild-type and *ppr40* mutant plants in well-watered and water-stressed conditions. Plants were cultured in growth chambers in standard conditions for 4 weeks; then watering was suspended for 10 days. Malondialdehyde (MDA) levels are shown in response to gradual stress. **b** Expression of the ROS-induced *ZAT12* gene in drought-stressed plants after 8 days of treatment. Relative transcript levels are shown, where 1 corresponds to values measured in Col-0 plants under control conditions. Reference: *Actin2* and *UBC18* genes. Averages of three replicates are shown, with standard deviation indicated by the bars on the diagrams. Statistical analysis used Two-way ANOVA, Tukey test. Different letters indicate significant differences at *P* < 0.05

Generation of reactive oxygen species (ROS) in higher plants is a common consequence of environmental stresses. Although ROS can be produced by various metabolic processes, in illuminated plants photosynthesis is the main source of superoxide, which is rapidly converted to hydrogen peroxide. Oxidative stress is the consequence of elevated ROS content and includes peroxidation of various types of macromolecules as well as lipids (Møller et al. 2007). As photosynthetic parameters were differently affected by water deprivation in wild-type and *ppr40* mutant plants, we decided to monitor oxidative damage during water stress. Lipid peroxidation was estimated by measuring malondialdehyde (MDA) levels, which increased with time in response to water stress, consistent with the notion that abiotic stress can trigger lipid peroxidation and oxidative damage. Lipid peroxidation rates started to increase after 7th day of water deprivation. In the following days MDA accumulated faster and reached 2 to 2.5 times higher level in Col-0 plants than in *ppr40-1*. Lipid peroxidation increased more slowly in the *ppr40-2* mutant than in wild-type plants, but it reached comparable level to Col-0 after nine days of water deprivation (Fig. 9a). Our results suggest that drought-dependent oxidative damage can be significantly lower in *ppr40-1* than in *ppr40-2* and wild-type plants.

ROS and ABA signals are known to interact and modulate stress responses, including activation of a set of stress-induced genes (Mittler and Blumwald 2015). Differences in lipid peroxidation rates suggested that ROS-induced gene expression might also be altered in the *ppr40* mutants. ZAT12 is a well-known transcription factor, which regulates the expression of a number of ROS-induced genes many of them encoding antioxidant proteins (Davletova et al. 2005). Transcript levels of *ZAT12* were enhanced by water deficiency in all genotypes, but the induction was lower in *ppr40* mutants, particularly in *ppr40-1* (Fig. 9b).

### Gene expression induced by water deficit is modulated by PPR40

**Fig. 10:**
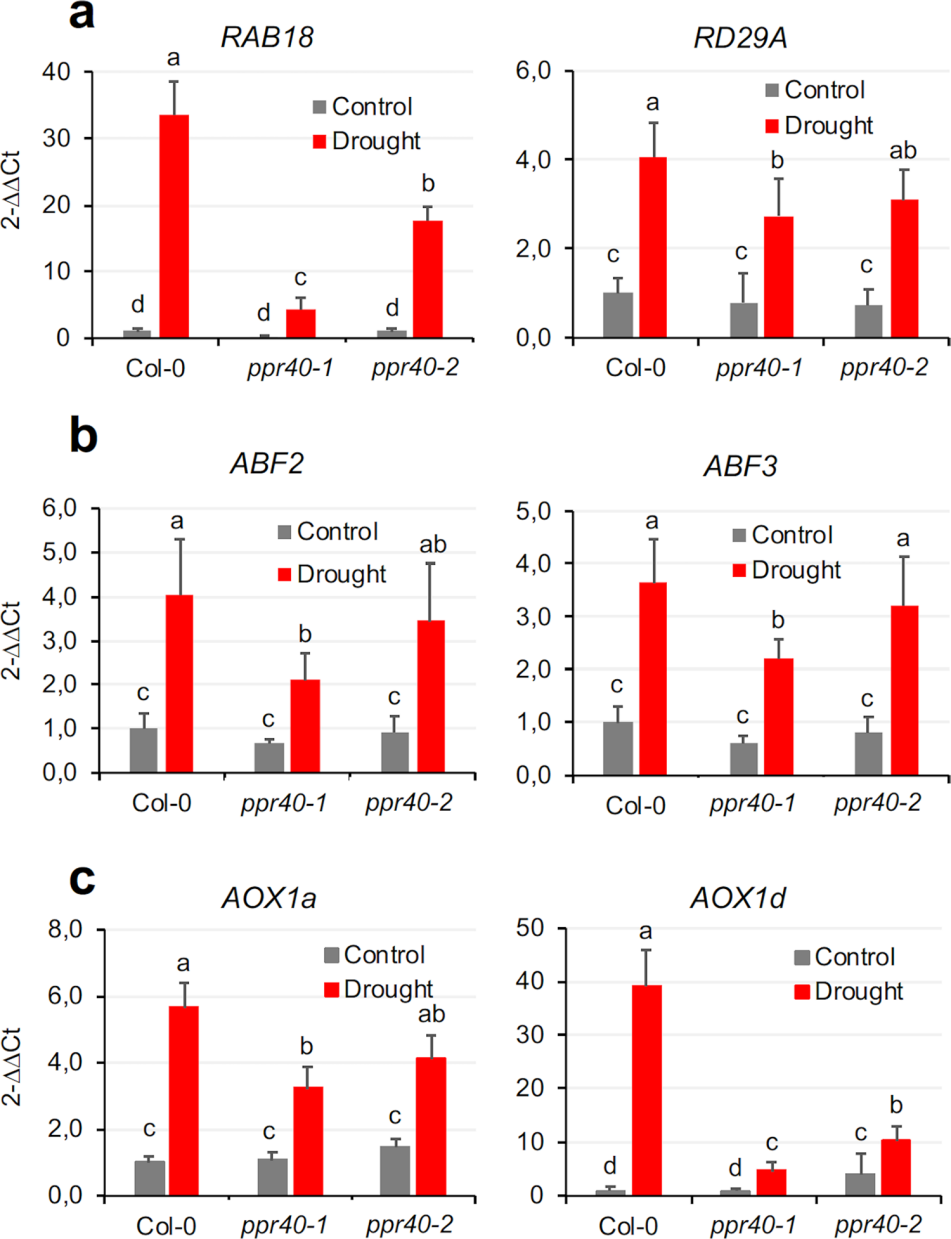
Expression of selected stress-induced genes in water-limited and well-watered conditions. Plants were grown and treated as indicated in Fig. 8. Transcript levels of *RD29A RAB18* (**a**)*, ABA-responsive element-binding factor 2* (*ABF2*)*,* and *ABF3* (**b**), and *AOX1a, AOX1d* (**c**) genes were measured by qRT-PCR using *Actin2* and *UBC18* as reference genes. Relative expression levels are shown, with a value of 1 corresponding to transcript levels of Col-0 in control plants. The data represent the average of three replicates, with standard deviation indicated by the bars on the diagrams. Statistical analysis used Two-way ANOVA, Tukey test. Different letters indicate significant differences at *P* < 0.05

Water deprivation leads to profound changes in gene expression profiles, as hundreds of genes are up- or downregulated in dehydrated plants. ABA is the most important stress hormone, which not only promotes fast physiological responses such as stomata closure in high osmotic conditions but also induces the expression of large set of dehydration-induced genes (Finkelstein 2013). To test the effect of the *ppr40* mutations on stress and ABA-dependent regulation, transcript levels of selected stress-induced genes were monitored in plants under water-limited conditions. Expression of the ABA-induced *RAB18* was enhanced 40 times by water stress in wild-type plants, which was induced only 3 and 20 times in *ppr40-1* and *ppr40-2* mutants, respectively. The drought-responsive *RD29A* was moderately induced in water-stressed plants, and the difference between the wild-type and mutant plants was less accentuated (Fig. 10a). The ABA-induced ABF transcription factors control the expression of many drought-responsive genes in plants (Finkelstein 2013). Under well-watered conditions, expression of *ABF2* and *ABF3* was similar in wild-type plants and mutants. In water-stressed plants transcript levels both genes increased three to four times in Col-0 plants but only twice in the *ppr40-1* mutant (Fig. 10b). Plant alternative oxidases are encoded by a small, stress-responsive *AOX* gene family (Clifton et al. 2006). The *AOX1a* and *AOX1d* genes responded to water stress in wild-type plants, but were less activated in the *ppr40* mutants than in wild-type plants. Difference was particularly notable in case of *AOX1d* expression, which is a dominant stress-induced *AOX* gene (Fig. 10c). These results indicate that drought-responsive transcriptional activation is less efficient in the *ppr40* mutants, particularly in *ppr40-1*.

ROS can modulate various hormone signaling pathways. In order to trace the possible influence of the *ppr40* mutations on hormone signaling, transcript levels of different hormone-regulated genes were compared in *ppr40-1* and *ppr40-2* mutants to Col-0 in standard or water-restricted conditions (Fig. S5). Transcript levels of *LAX3* (*Like Aux1 3*) and *AUX1,* implicated in auxin signaling, were repressed by water deprivation in wild-type plants, but were not or less inhibited in the *ppr40* mutants. In wild-type plants expression of the salicylic acid (SA) responsive *NPR1* (*Non-expresser of PR genes 1*) was reduced to half, but was not affected in the *ppr40* mutants. *WRKY30* (*WRKY DNA-binding protein 30*) is regulated by ethylene, jasmonate, and brassinosteroids which was downregulated in water-deprived wild-type plants, but was only slightly induced in *ppr40* mutants. *EIN3* (*Ethylene-insensitive 3*) is implicated in ethylene and jasmonate signaling whose expression was unaffected by the *ppr40* mutations. Transcript levels of the jasmonate-regulated *COI1* (*Coronatine-insensitive 1*) were moderately enhanced by water stress, with no or only minor differences in *ppr40* mutants. Results of the transcript analysis suggest that PPR40 has influence not only on ABA-dependent regulation, but can modulate pathways controlled by other hormones such as auxin, salicylic acid, and brassinosteroids.

## Discussion

Our earlier data revealed that the mitochondria-localized PPR40 protein has direct effect on cellular respiration and it is involved in salt, osmotic, and oxidative stress responses (Zsigmond et al. 2008). Most PPR proteins can bind RNA through their conserved PPR domains and are implicated in splicing or editing RNAs encoded in chloroplasts or mitochondria (Barkan and Small 2014). Similar to PPR40, several PPR proteins were shown to modulate stress responses. Such reports, however, describe that these proteins are implicated in post-transcriptional processing of organelle-encoded RNA sequences (Yuan and Liu 2012; Zhu et al. 2014; Qiu et al. 2021). PPR40 does not bind RNA, neither is involved in RNA processing (Zsigmond et al. 2008). Nevertheless, the PPR40 protein can stabilize the mitochondrial electron transport and reduce the amount of ROS generated under salt stress (Zsigmond et al. 2008, 2012). The absence of PPR40 protein in *ppr40-1* compromised mitochondrial electron transport through Complex III leading to enhanced ROS generation, higher H_2_O_2_ content, and hypersensitivity to ABA, but no ABA content (Zsigmond et al. 2008). Hydrogen peroxide was shown to mediate ABA signals in guard cells and together with cytosolic Ca^2+^, it functions as second messenger in coordination of ion transport which is needed for stomata closure (Zhang et al. 2001; Kwak et al. 2003; Postiglione and Muday 2020). Activation of NADPH oxidases (Respiratory Burst Oxidase Homologs, RBOH) by SnRK2-mediated phosphorylation can control ROS accumulation in response to ABA (Kwak et al. 2003; Sirichandra et al. 2009). An alternative ROS-generating pathway has recently been described, which suggests that mitochondria and chloroplasts are important sources of ABA-generated H_2_O_2_ in guard cells. Mitochondria-derived H_2_O_2_ was demonstrated to be responsible for enhanced ABA sensitivity and to promote ABA-triggered stomata closure (Postiglione and Muday 2023). ABA contents of *ppr40-1* and wild-type plants were similar (Zsigmond et al. 2008), suggesting that difference in ABA responses could be responsible for the observed responses during water deprivation. Enhanced ABA sensitivity of the *ppr40-1* mutant can be derived from the increased H_2_O_2_ production of the damaged mitochondrial electron transport which can therefore promote stomata closure (Zsigmond et al. 2008). Guard cells of *ppr40-1* could be particularly responsive to ABA due to the mitochondria-derived peroxide signals (Postiglione and Muday 2020, 2023). More closed stomata could contribute to reduced evaporation and better water retention in the mutants (Fig. 4), which could have far-reaching physiological consequences in water-limiting environment.

Mitochondria can influence a number of metabolic and hormonal signaling pathways and modulate physiological processes, ROS homeostasis, and gene expression patterns (Rhoads and Subbaiah 2007; Møller et al. 2021). Several mutants with mitochondrial defects were shown to display altered ABA sensitivity, especially during germination which is a particularly energy-demanding process (Wang et al. 2014; Huang et al. 2016). Mitochondrial ROS have been shown to affect plant hormone signaling, in particular, abscisic acid and auxin. The *hot ABA-deficiency suppressor2* (*HAS2*) encodes a Pentatricopeptide repeat (PPR) protein which is needed for editing a cytochrome c-heme lyase subunit coding RNA. The *has2* mutation was shown to cause increased respiration, accelerate H_2_O_2_ production, and enhance ABA sensitivity of stomata closure leading to increased drought tolerance (Sechet et al. 2015). SLG1 is a PPR protein, which is needed for RNA editing of nad3, a key component of Complex I in mETC. Mitochondria in the *slg1* mutant has impaired electron transport producing excess H_2_O_2,_ which generates ABA hypersensitivity, faster stomata closure, enhancing tolerance to water deprivation (Yuan and Liu 2012). Both *slg1* and *ppr40-1* mutants were characterized by slow growth and delayed development. Impairment of mETC by mutations which disrupt either Complex I or Complex III seems to generate similar phenotypes and enhance ABA responses with beneficial effect on drought tolerance. Impaired stomatal conductance can be the consequence of ABA hypersensitivity and more closed stomata, which may limit CO_2_ fixation in photosynthetic leaves. Semidwarf phenotype of the *ppr40-1* mutant apparently correlates with such reduced CO_2_ fixation rates and can be the consequence of it.

Due to faster stomatal closure, water loss is contained, the leaf water content of drying plants remains higher, which can reduce osmotic and oxidative stress (Bharath et al. 2021). ABA hypersensitivity improved the capacity of the *ppr40-1* and to a lesser extent the *ppr40-2* mutant to preserve water in limiting conditions. Detailed phenotypic, physiological, and molecular analysis revealed that *ppr40-1* mutant has more robust, while *ppr40-2* has moderate tolerance to water deficiency when compared to wild-type plants, suggesting that *ppr40-1* is a stronger, while *ppr40-2* is a weaker mutant allele. Higher water content in water-limited conditions can influence various physiological and molecular parameters which can affect tolerance of stressed plants. Expression of a set of drought and ABA-induced genes was less induced by water deprivation in the *ppr40* mutants than in wild-type plants. *RAB18* is an ABA-induced dehydrin gene, which is upregulated during seed desiccation as well as in dehydrated plants. *RD29A* is another drought-responsive gene which is regulated by both ABA-dependent and independent signals. The bZIP-type ABF transcription factors are the main regulators of ABA-induced transcription and are encoded by a small gene family, whose members are upregulated by ABA (Finkelstein 2013). Difference in their expression in the *ppr40* mutants can be the consequence of the hydration status during water deficiency, as indicated by RWC data. Reduced water loss in *ppr40-1* alleviated dehydration leading to contained osmotic stress and ROS accumulation, which in turn provoked minor stress-responsive gene activation.

Proline accumulation is a characteristic metabolic response to high osmotic conditions and was attributed to have protective effect (Székely et al. 2008; Alvarez et al. 2022). In response to water deficiency proline accumulation in *ppr40-1* lagged behind *ppr40-2* and the wild-type plants, which could be attributed to higher RWC and inferior P5CS1 activation of these plants in such conditions (Fig. 5). Negative correlation between leaf water content and proline accumulation has previously been described which agrees with our results (Sperdouli and Moustakas 2012). Transcript levels of the ABA-induced *P5CS1* gene were lower in the *ppr40* mutants than in Col-0 plants under water deprivation. *P5CS1* controls the rate-limiting reaction in proline biosynthesis during stress and the difference in expression correlated with the inferior proline accumulation. Besides proline metabolism many other metabolic pathways are modulated by water deprivation. Stress-induced shifts of various amino acids, organic acids, and sugar derivatives were attenuated in the *ppr40* mutants (Fig. 6), indicating that stress-induced physiological changes are less robust in these mutants. Alterations of citric acid and fumaric acid concentrations in water-deprived *ppr40-1* plants were less drastic than in wild-type plants, suggesting that the TCA cycle in mutant mitochondria could be sustained better in such conditions.

Photosynthesis is one of the metabolic processes which is most sensitive to drought. The light-harvesting complex II (LHCII) is disintegrated, thylakoid membrane proteins are degraded, and chlorophyll content is reduced as a consequence of water stress (Chen et al. 2016). In our experiments, photosynthetic parameters such as PSII quantum yield or electron transport rates were drastically affected in water-stressed wild-type plants. When time course of Fv/Fm and QY-lss1 change was analyzed in the phenotyping system, decline of these parameters was slower in *ppr40-1* and *ppr40-2* mutants than in Col-0 wild-type plants (Fig. 7, Fig. S4). Not only the maximum PSII quantum yield was more stable in the mutants, but also the electron transport rate of PSII, measured at different light intensities, remained significantly higher in *ppr40-1* and in less degree in *ppr40-2* (Fig. 8). These data suggest that drought-generated photo-damage of PSII can be alleviated in *ppr40* mutants. Such observations correlated with the attenuated oxidative damage of the mutants (Fig. 9).

PSII in the chloroplast is the dominant site for ROS generation, producing singlet oxygen, superoxide anion, and hydrogen peroxide in stress conditions such as drought (Chen et al. 2016; Ruban et al. 2012). Singlet oxygen and hydrogen peroxide can trigger oxidative damage of different molecules and cellular structures and enhance lipid peroxidation (Noctor et al. 2018). The degree of cell damage caused by oxidative stress correlates with lipid peroxidation rates, which increased with time during dehydration and was significantly lower in *ppr40-1* mutant than in wild-type or *ppr40-2* plants (Fig. 9), correlating with the more stable photosynthetic processes. H_2_O_2_ is not only a cell-damaging molecule but also an important stress signal, which can trigger defense-related reactions including activation of a set of target genes (Miller et al. 2010). The zinc-finger transcription factor ZAT12 is a key regulator of ROS signaling and the *ZAT12* gene is induced by various stress conditions as well as by ROS (Davletova et al. 2005). As expected, *ZAT12* was induced by water deficiency in wild-type plants, but its activation was inferior in the *ppr40* mutants, in particular, in *ppr40-1* (Fig. 9). Less efficient activation of *ZAT12* was consistent with the reduced oxidative damage and moderate inhibition of photosynthetic processes in *ppr40-1*. ROS signals control a number of developmental and defense-related pathways, which are controlled by different hormones. PPR40 might therefore influence expression of genes which are implicated in hormonal regulation. *LAX3* and *AUX1* are implicated in auxin signal transduction (Péret et al. 2012). Non-expresser of PR genes 1 (NPR1) is a master regulator of salicylic acid (SA) signals in pathogen response pathways (Chen et al. 2021). WRKY DNA-binding protein 30 (WRKY30) is implicated in responses to different environmental stimuli and its activity is controlled by MAP kinases and various plant hormones, such as ethylene, jasmonate, brassinosteroids, and cytokinin (Jiang et al. 2017; Andrási et al. 2019). Ethylene-insensitive 3 (EIN3) is a key transcription regulator in ethylene signaling, which is implicated in jasmonate and wound response as well (Song et al. 2022). Coronatine-insensitive 1 (COI1) is part of the jasmonate receptor complex, a key player in wound and jasmonate-dependent signaling (Wasternack and Song 2017). Altered transcriptional response of auxin, SA, and JA-regulated genes to water deprivation suggests that PPR40-modulated ROS signals can influence not only ABA regulation but also other hormone-controlled pathways (Fig. S5).

Photosynthesis and mitochondrial respiration are in close interaction through various metabolic and signaling pathways (Blanco et al. 2014; Van Aken 2021). Plant growth depends on net carbon fixation which is defined by CO_2_ uptake by photosynthesis and CO_2_ release by mitochondrial respiration. In stress conditions mitochondria can influence photosynthetic processes in multiple way. Alternative oxidases (AOXs) are involved in the non-energy conserving electron transport pathway which helps maintain photosynthesis by stabilizing the energy balance in stress conditions such as drought (Vanlerberghe et al. 2016). When the cytochrome c pathway is inhibited by stress, AOX allows electron flow from the reducing equivalents produced by the TCA cycle and photosynthesis to O_2_, increasing mitochondrial electron transport flexibility. Thus, the stress-induced alternative respiratory pathway prevents excessive ROS production (Vishwakarma et al. 2015; Van Aken 2021). Plant AOX is encoded by a small gene family, which can respond to various stress conditions (Clifton et al. 2006). The absence of *AOX1a* results in decreased activity of photosynthesis and increased ROS accumulation in Arabidopsis (Giraud et al. 2008). *AOX1a* and *AOX1d* transcript levels were significantly higher in water-stressed wild-type plants, but were less enhanced in the *ppr40-1* mutant and intermediate in *ppr40-2* (Fig. 10C). Alternative oxidases (AOX) function as non-energy conserving terminal oxidases in the mitochondrial electron transport, which can alleviate over-reduction of the mETC and reduce oxidative damage during stress. Transcript data demonstrate that the stress-responsive non-phosphorylating respiratory pathway was less activated in the *ppr40* mutants, suggesting that these plants experienced limited stress.

## Conclusion

Our working hypothesis is summarized in a model which is depicted in Fig. S6. The improved tolerance of *ppr40-1* mutant plants to water deficit is reflected by various parameters including increased viability, contained growth defect, reduced oxidative damage, and sustained photosynthetic capacity. ABA hypersensitivity of *ppr40-1* and to less extent the *ppr40-2* mutants can be the primary reason of their tolerance to water deficiency, which influenced multiple metabolic and defense pathways. Our data suggest that the mitochondrial PPR40 can modulate regulatory mechanisms linking ABA and H_2_O_2_ signals and play important roles in regulating the molecular mechanisms of stress responses, which influence plant survival under water deficit. Precise understanding of the regulatory function of PPR40 protein, however, requires further studies.

### Supplementary Information

Below is the link to the electronic supplementary material.

Supplementary file1 (PDF 1393 KB)

## Data Availability

Data will be made available upon reasonable request.

## Abbreviations

AOX: Alternative oxidase
mETC: Mitochondrial electron transport chain
PDH: Proline dehydrogenase
P5CS: D-pyrroline carboxylate synthase
PPR: Pentatricopeptide repeat
RAB18: Responsive to ABA 18
RD29A: Responsive to desiccation 29A
RGB: Red–green–blue
ROS: Reactive oxygen species
RWC: Relative water content
SOL: Slenderness of leaves
TCA cycle: Tricarboxylic acid cycle
ZAT12: Zinc finger protein of Arabidopsis thaliana 12
