# Correction to: Mutation in Arabidopsis mitochondrial Pentatricopeptide repeat 40 gene affects tolerance to water deficit

**DOI:** 10.1007/s00425-024-04443-w

**Published:** 2024-05-27

**Authors:** Kamal Kant, Gábor Rigó, Dóra Faragó, Dániel Benyó, Roland Tengölics, László Szabados, Laura Zsigmond

**Affiliations:** 1 Institute of Plant Biology, HUN-REN Biological Research Centre, Temesvári Krt. 62, 6726 Szeged, Hungary; 2 Institute of Biochemistry, HUN-REN Biological Research Centre, Temesvári Krt. 62, 6726 Szeged, Hungary

**Correction to: Planta (2024) 259:78** 10.1007/s00425-024-04354-w

In this article the funding from 'Nemzeti Kutatási, Fejlesztési és Innovációs Hivatal (NKFIH)' was omitted. The complete funding section should read as below.

**Funding**

Open access funding provided by HUN-REN Biological Research Centre, Szeged. This research was supported by research grants of Nemzeti Kutatási, Fejlesztési és Innovációs Hivatal (NKFIH) K128728, FK128920, K143620, GINOP-2.3.3-15-2016-00023.

