# Supplementary material for: Mutation in Arabidopsis mitochondrial Pentatricopeptide repeat 40 gene affects tolerance to water deficit
Source: Planta. 2024 Mar 1;259(4):78. doi: 10.1007/s00425-024-04354-w (PMC10907415; doi:10.1007/s00425-024-04354-w)
Supplement: Supplementary file 1 — Supplementary file1 (PDF 1393 KB) [file 425_2024_4354_MOESM1_ESM.pdf]

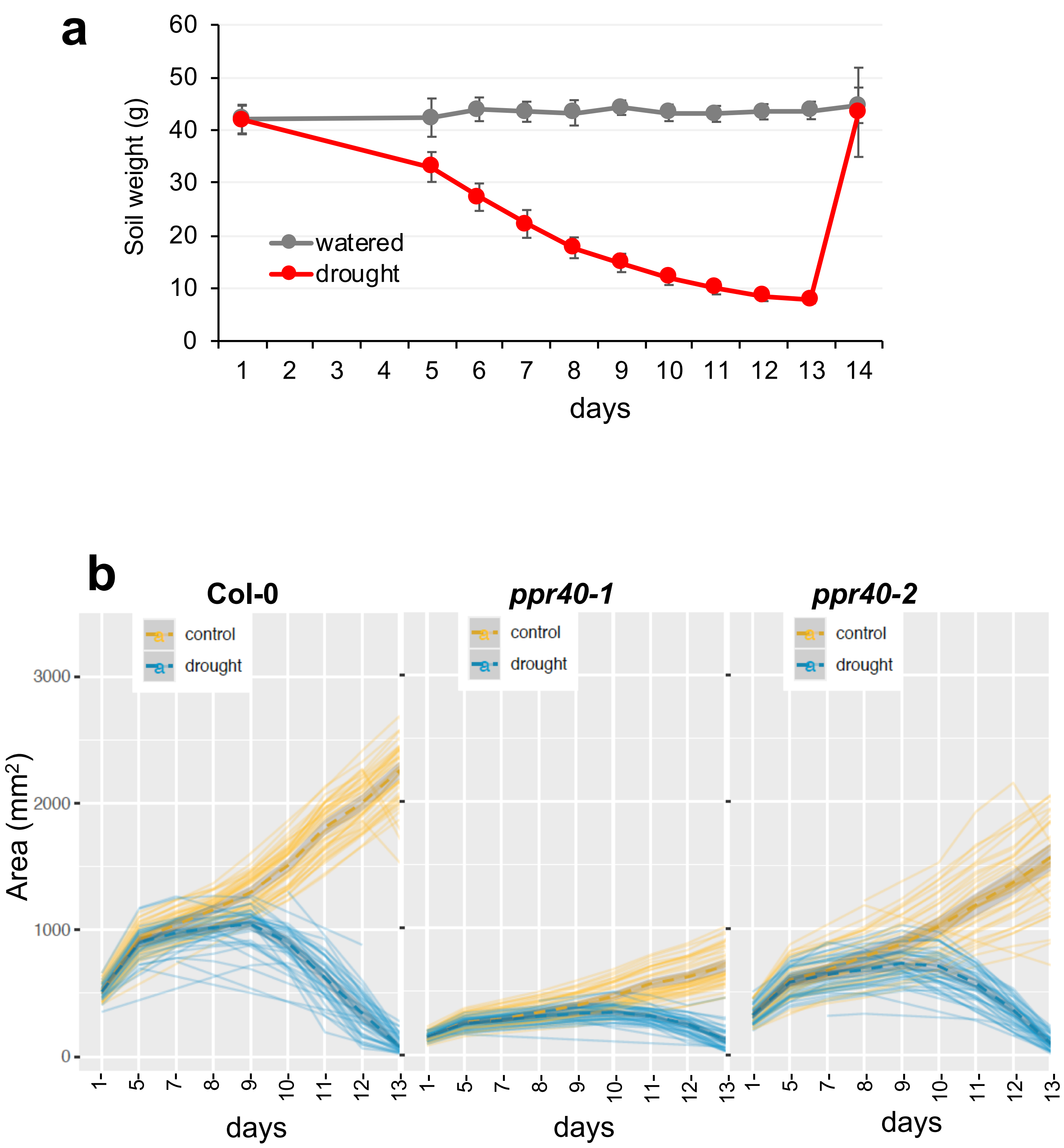

Figure S1. Plant phenotyping experiment. A) Change of soil weight in water deprivation experiment. B) Rosette areas of Col-0, *ppr40-1* and *ppr40-2* plants in the plant phenotyping experiment. Yellow lines indicate plants in well-watered conditions, blue lines show plants with drought treatment. Watering was suspended at day 1.

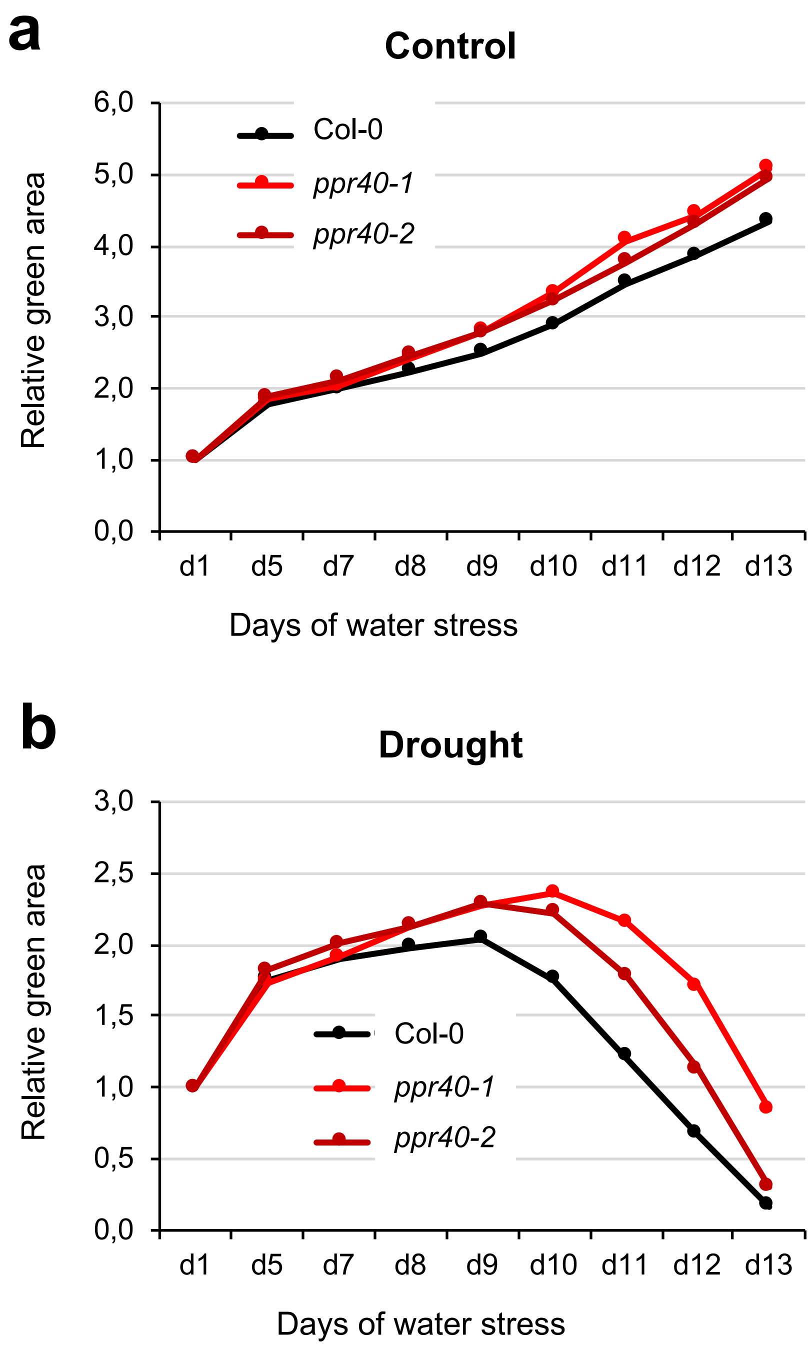

Figure S2. Change of rosettes sizes, displayed as relative green areas of Col-0*, ppr40-1* and *ppr40-2* plants, determined by plant phenotyping. Relative values are shown, which are normalized to the first day of the imaging period which started when watering was suspended (day 1).

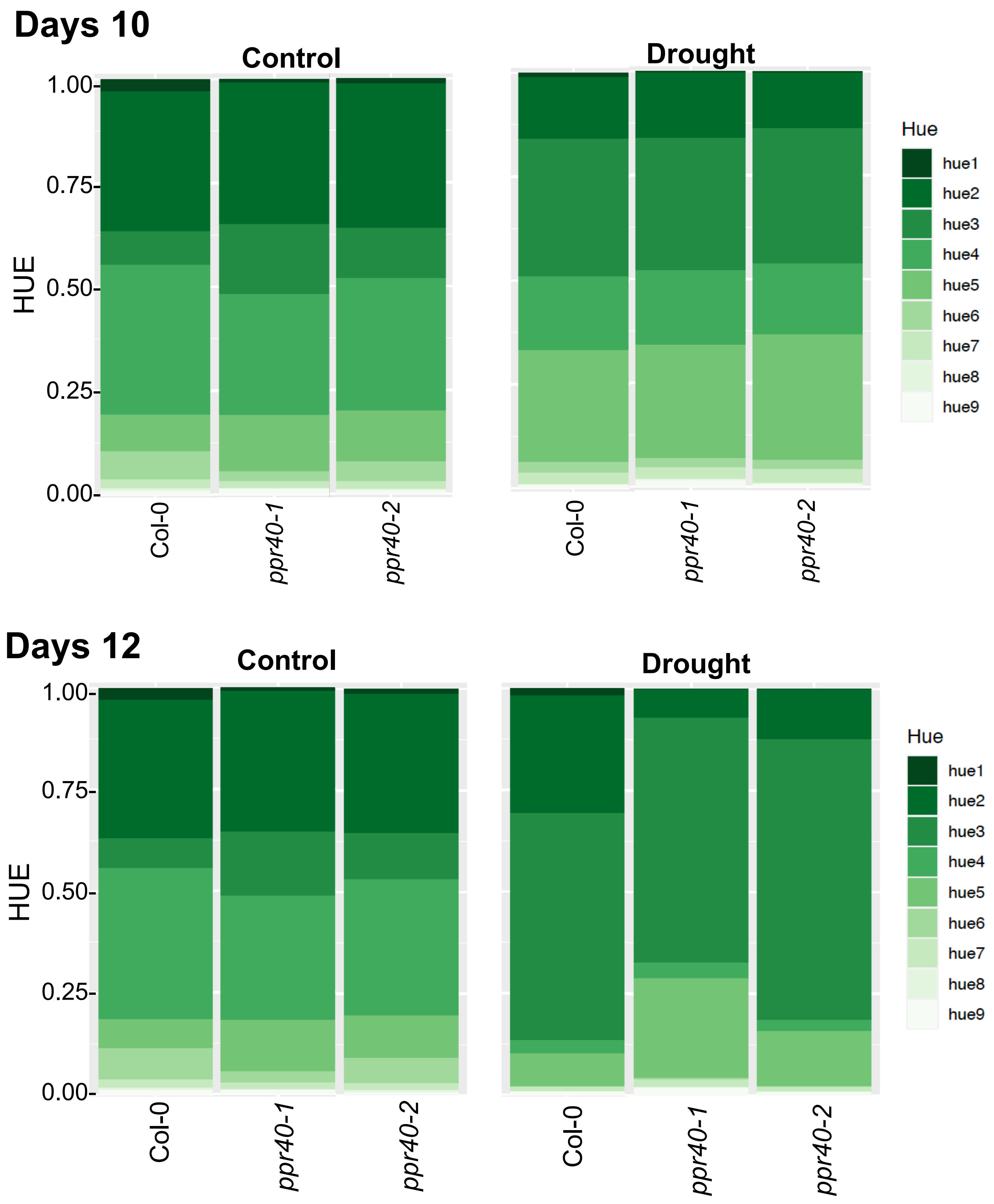

Figure S3. Change of greenness hue index of Col-0, *ppr40-1* and *ppr40-2* plants after 10 and 12 days of drought as detected by RGB images in the plant phenotyping experiment.
RGB images were segmented according to Awlia et al., (2016).

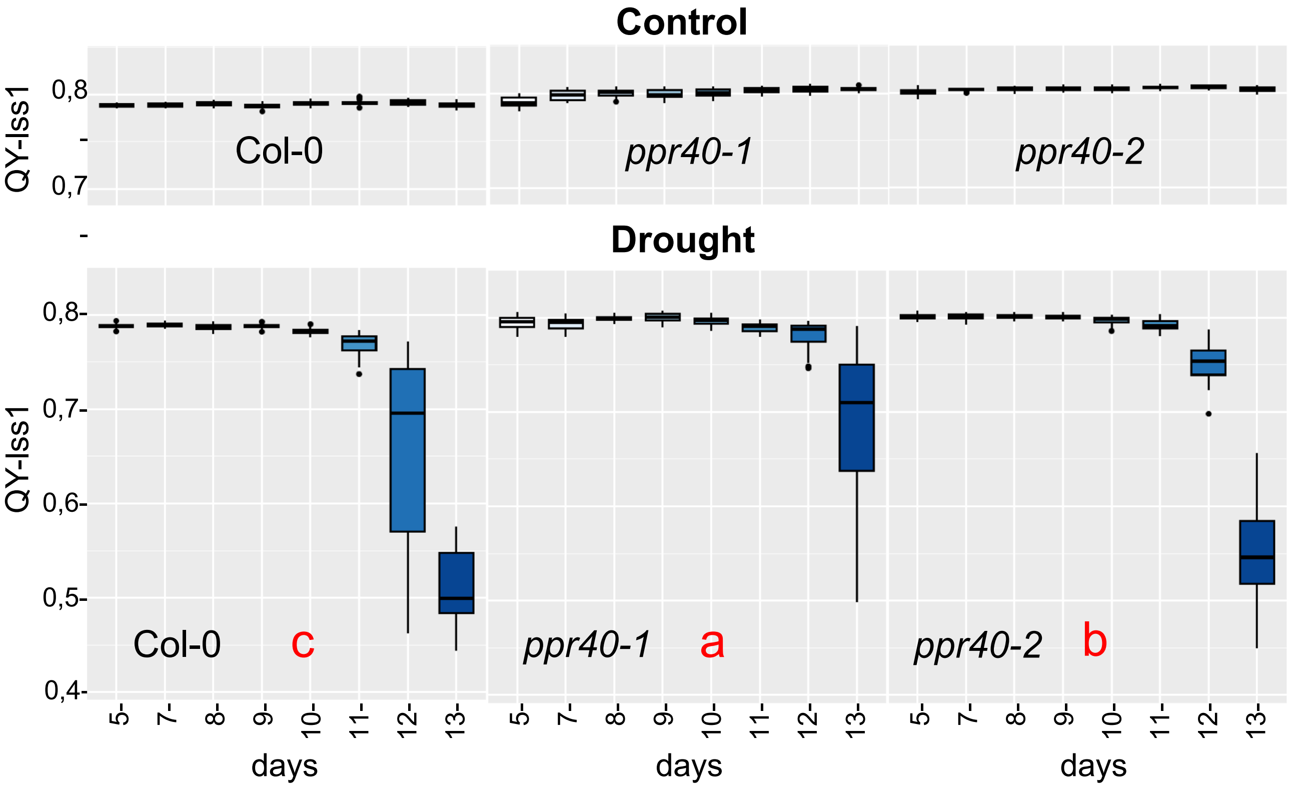

Figure S4. Photosynthetic parameters in well-watered and drought-stressed plants determined by ChlF imaging in an automatic phenotyping system. Change of QY-Iss1 in Col-0, *ppr40-1* and *ppr40-2* plants is shown 5 to 13 days after watering was stopped .

Control: uninterrupted watering; Drought: water withdrawal. Statistical analysis used Kruskal-Wallis test, to compare the treatments and genotypes. Different letters (in red) indicate significant differences at $p < 0.05$.

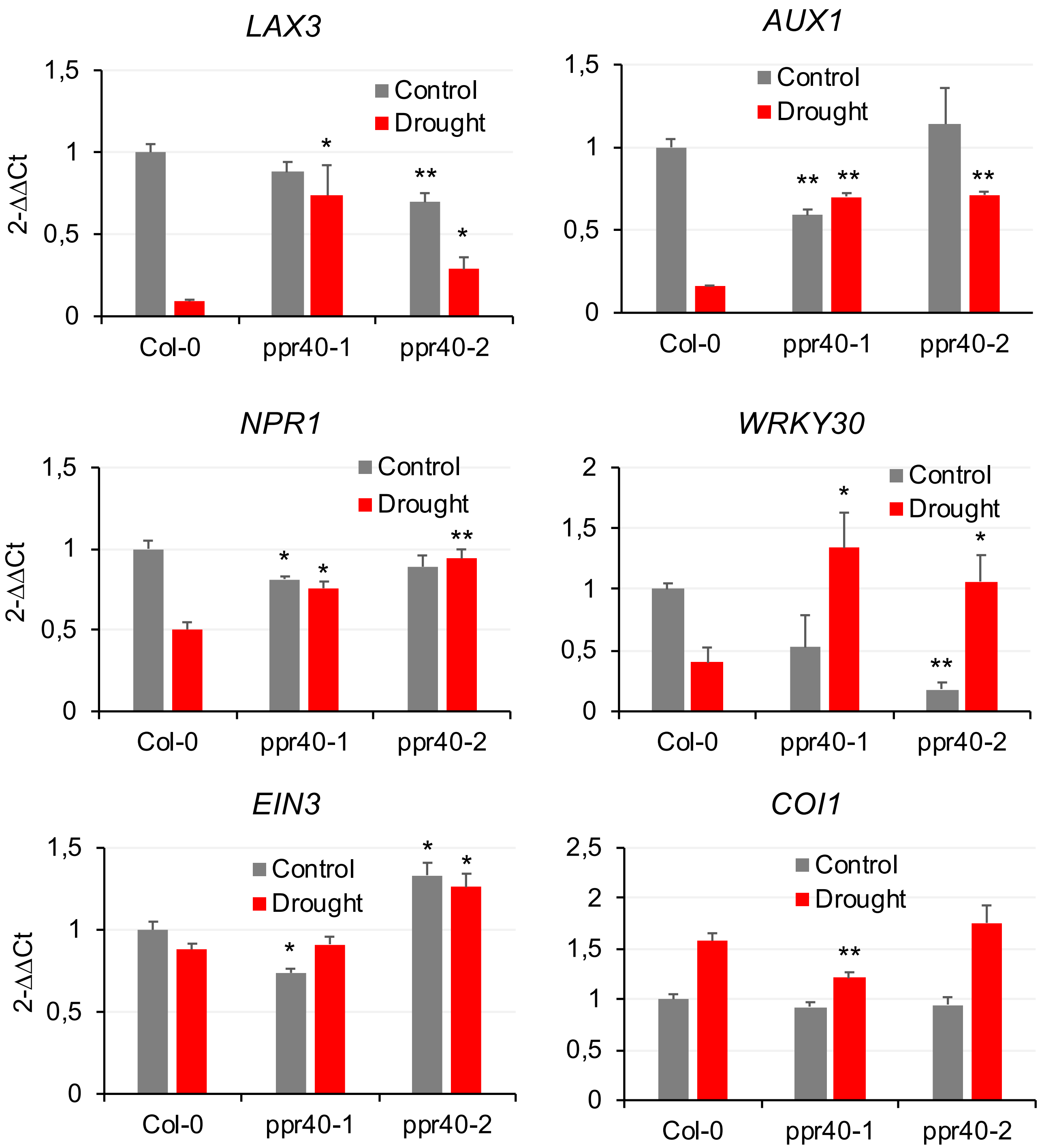

Figure S5. Expression profiles of various hormone-regulated genes in Col-0 wild type, *ppr40-1* and *ppr40-2* mutant plants. Plants were subjected to water stress and transcript levels were determined as indicated in Figure 9. Relative expression levels of *LAX3, AUX1, NPR1, WRKY30, EIN3* and *COI1* genes are shown, where 1 corresponds to transcript levels of Col-0 in control plants.

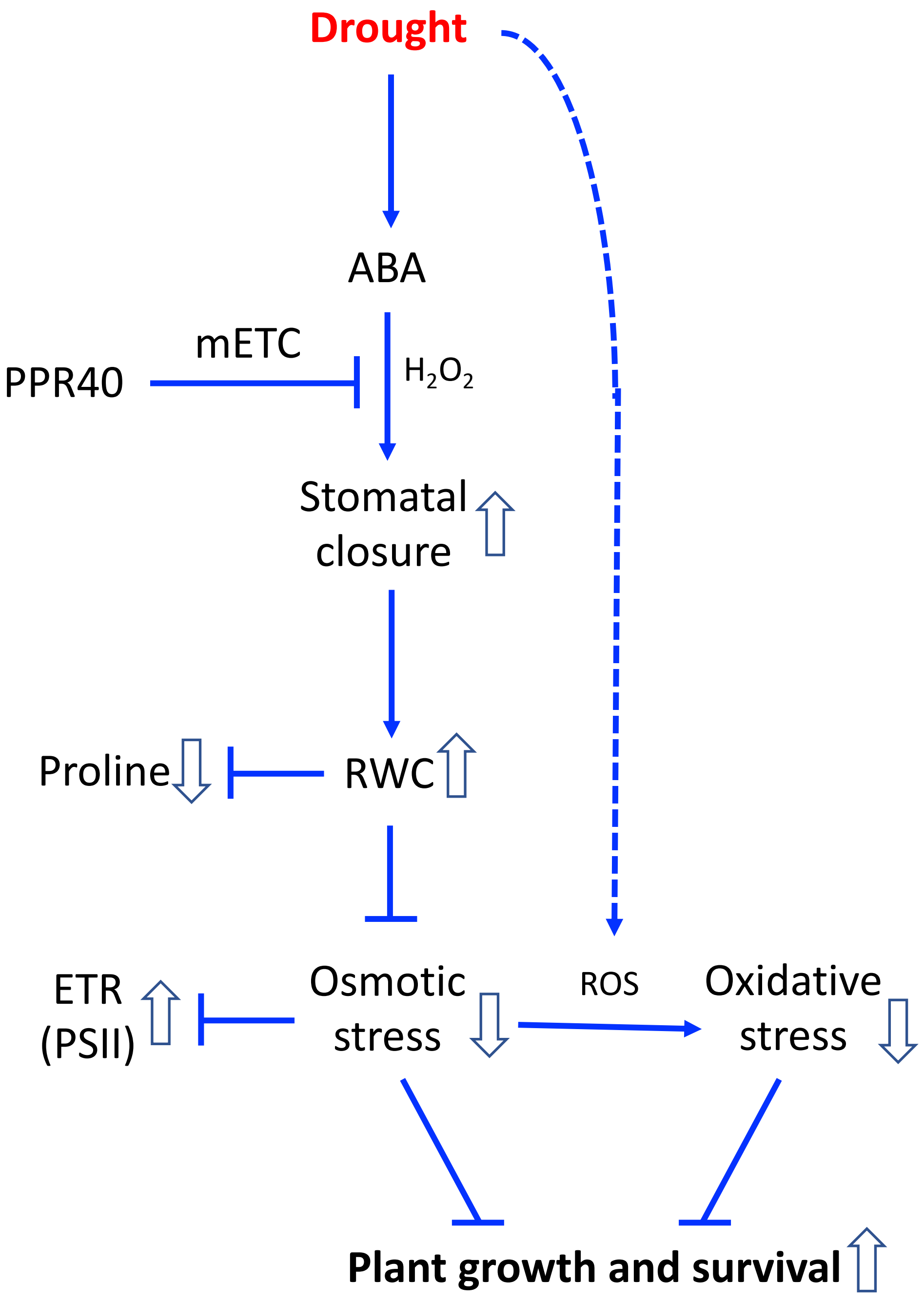

Figure S6.

Model of PPR40 action in drought responses of Arabidopsis plants. PPR40 is associated with Complex III in mETC and can downregulate ABA signaling in dehydrating plants as it stabilize electron transport and suppress ROS generation. ABA-induced stomata closure is faster in the *ppr40-1* mutant reducing water loss during drought and leading to higher RWC that diminishes osmotic stress. Reduced proline accumulation in *ppr40-1* can be the consequence of higher RWC and lower osmotic pressure. Lower osmotic and oxidative stress in drought-stressed *ppr40-1* can alleviate inhibition of photosynthetic ETR and contribute to enhanced viability and growth of drought-stressed *ppr40-1* plants. Small arrows indicate enhanced or diminished features in water-stressed *ppr40-1* mutant.

Table S1. List of oligonucleotides used in this study

Gene	Locus	Forward Primer (5'-3')	Reverse Primer (5'-3')	Reference
ACT2	At3g18780	GGTAACATTGTGCTCAGTGGTGG	AACGACCTTAATCTTCATGCTGC	An YQ et al. 1996
UBC18	At5g42990	ACAGCAATGGACATATTTGTTTAGA	TGATGCAGACTGAACTCACTGTC	Ayaydin F. et al. 2015
ZAT12	At5g59820	GACGCTTTGTCGTCTGGATT	GTGTCCTCCCAAAGCTTGTC	Perez-Salamó et al. 2014
RD29A	At5g52310	ATCACTTGGCTCCACTGTTGTTC	ACAAAACACACATAAACATCCAAAGT	Pandey et al. 2004
RAB18	At5g66400	CAGCAGCAGTATGACGAGTA	CAGTTCCAAAGCCTTCAGTC	Pandey et al. 2004
P5CS1	At2g39800	GGCAGATGGTCTTGTCTTAGAG	CACTACGGATGGCAAGTGAA	in this study
PDH1	At3g30775	GTCCTCTCTACCACACAAACTC	TGGACTCTTGGCATTTCCTAC	in this study
AOX1A	At3g22370	CTGGACCACGTTTGTTC	ACACCCCAATAGCTCG	Saisho et al. 1997
AOX1D	At1g32350	ATTGGAGGATTCAGGGGACA	CGTTCGGATAGGATTTTCTGG	in this study
AUX1	At2g38120	CTTTCCTCCTCTGCACATTTCT	AAGAGTGGTTTTTGTCCGTTTG	Rigó et al., 2013
LAX3	At1g77690	TGCTTACCTTTGCTCCTGCT	GTCCCCATCCATCCTCCTAC	Rigó et al., 2013
EIN3	At3g20770	AACTTTGGGATGGTTGCTAAAA	CTGGGACTTCTTCTTTGACAGG	in this study
WRKY30	At5g24110	AGAGCGATGATTCCGATCAAG	CATCGTCCAGCGTTCTATCAA	Besseau et al., 2012
COI1	At2g39940	TGATGATGTCATCGAGCAAG	ATGCTCTCTCGTCTCGGAAT	Liu et al., 2016
NPR1	At1g64280	TTTACAGCAGCAGAGTGAGACC	AGCCAAATAGAGAACCTCCAACA	in this study

References:

An YQ, McDowell JM, Huang S, McKinney EC, Chambliss S, Meagher RB (1996)Strong, constitutive expression of the Arabidopsis ACT2/ACT8 actin subclass in vegetative tissues. Plant J 10: 107-121.

Ayaydin F, Bíró J, Domoki M, Ferenc Gy, Fehér A (2015) Arabidopsis NAP-related proteins (NRPs) are soluble nuclear proteins immobilized by heat. Acta Physiol Plant (2015) 37:3.

Pandey S, Assmann SM (2004) The Arabidopsis putative G protein-coupled receptor GCR1 interacts with the G protein alpha subunit GPA1 and regulates abscisic acid signaling. Plant Cell 16: 1616-1632.

Perez-Salamo, I., Papdi, C., Rigo, G., Zsigmond, L., Vilela, B., Lumbreras, V., Nagy, I., Horváth, B., Domoki M., Darula, Z., Medzihradszky, K., Bögre, L., Koncz, C., Szabados, L. (2014). The Heat Shock Factor A4A Confers Salt Tolerance and Is Regulated by Oxidative Stress and the Mitogen-Activated Protein Kinases MPK3 and MPK6. PLANT PHYSIOLOGY, 165(1), 319–334.

Saisho D, Nambara E, Naito S, Tsutsumi N, Hirai A, Nakazono M (1997) Characterization of the gene family for alternative oxidase from Arabidopsis thaliana. Plant Mol Biol 35: 585-596.
